# Downgrading disease transmission risk estimates using terminal importations

**DOI:** 10.1371/journal.pntd.0007395

**Published:** 2019-06-14

**Authors:** Spencer J. Fox, Steven E. Bellan, T. Alex Perkins, Michael A. Johansson, Lauren Ancel Meyers

**Affiliations:** 1 Department of Integrative Biology, University of Texas at Austin, Austin, Texas, United States of America; 2 Department of Epidemiology and Biostatistics, University of Georgia, Athens, Georgia, United States of America; 3 Center for Ecology of Infectious Diseases, University of Georgia, Athens, Gerogia, United States of America; 4 Department of Biological Sciences and Eck Institute for Global Health, University of Notre Dame, Notre Dame, Indiana, United States of America; 5 Division of Vector-Borne Diseases, Centers for Disease Control and Prevention, San Juan, Puerto Rico; 6 Center for Communicable Disease Dynamics, Harvard TH Chan School of Public Health, Boston, Massachusetts, United States of America; 7 Santa Fe Institute, Santa Fe, New Mexico, United States of America; Imperial College London, UNITED KINGDOM

## Abstract

As emerging and re-emerging infectious arboviruses like dengue, chikungunya, and Zika threaten new populations worldwide, officials scramble to assess local severity and transmissibility, with little to no epidemiological history to draw upon. Indirect estimates of risk from vector habitat suitability maps are prone to great uncertainty, while direct estimates from epidemiological data are only possible after cases accumulate and, given environmental constraints on arbovirus transmission, cannot be widely generalized beyond the focal region. Combining these complementary methods, we use disease importation and transmission data to improve the accuracy and precision of *a priori* ecological risk estimates. We demonstrate this approach by estimating the spatiotemporal risks of Zika virus transmission throughout Texas, a high-risk region in the southern United States. Our estimates are, on average, 80% lower than published ecological estimates—with only six of 254 Texas counties deemed capable of sustaining a Zika epidemic—and they are consistent with the number of autochthonous cases detected in 2017. Importantly our method provides a framework for model comparison, as our mechanistic understanding of arbovirus transmission continues to improve. Real-time updating of prior risk estimates as importations and outbreaks arise can thereby provide critical, early insight into local transmission risks as emerging arboviruses expand their global reach.

## Author summary

When novel infectious diseases like chikungunya or Zika emerge and threaten global spread, public health officials worldwide must assess the risk for local introductions and outbreaks. These assessments are made in anticipation of local case data, and officials must draw upon historic evidence from similar diseases or locations. Thus, accurate local risk assessments have most often been limited to retrospective analyses and have been unavailable in real-time during an emerging epidemic. Here we present a method that can harness both historic and current data to produce early risk assessments and update projections in real-time. We demonstrate our approach by estimating local transmission risk for Zika throughout the state of Texas. Our findings suggest that the majority of Texas counties face little risk for sustaining a Zika epidemic, and successfully predict the number of locally transmitted cases across the state. Real-time updating of local transmission risk estimates during an emerging epidemic can thus provide actionable, early insight for public health response as emerging arboviruses expand their global reach.

## Introduction

The explosive emergence of Zika virus (ZIKV) in the Americas in 2016 caught the global health community by surprise. Officials scrambled not only to control the disease at its source but also to anticipate and rapidly contain global transmission via infected travelers [1]. The rate at which a newly introduced pathogen spreads can vary enormously though, particularly for ZIKV and other pathogens with multi-faceted drivers of their transmission [2]. For example, serological surveys of human exposure to the ecologically similar dengue virus (DENV) on either side of the Texas-Mexico border indicated far higher DENV exposure in the Mexican community, despite virtually identical climatic conditions and even higher mosquito abundance in the Texan community [3]. This result suggests that *a priori* prediction of risk may be quite challenging.

Epidemiological risk assessment—estimating the severity and transmissibility of a threatening disease—can be vital to successful mitigation with limited resources. Historical outbreak data can provide invaluable insight into future epidemic risk. However, for a pathogen that has yet to arrive or has just begun to spread, we are forced to borrow epidemiological data from other populations or related pathogens, or to indirectly assess risk based on environmental suitability. For example, as the first importations of ZIKV arrived in the US in 2016, early attempts to determine the likelihood and rate of local transmission relied primarily on dengue epidemiological data from regions with markedly different climatic and socioeconomic conditions [4–6].

These ecological risk assessments provide information regarding the basic reproduction number of a disease (*R*_0_)—the expected number of secondary human infections resulting from a single human infection—which provides a meaningful and predictive measure of local epidemiological risk. For ZIKV, *R*_0_ encompasses the combined effects of the multi-step transmission life cycle: a mosquito biting an infected individual, incubating the disease and becoming infected, and finally biting and infecting a new susceptible individual. In a naive population, *R*_0_ indicates whether importations can potentially lead to sustained local epidemics; if so, it also provides insight into the probability, magnitude, and speed of spread [7, 8]. However, ecological estimates often carry considerable uncertainty stemming from model parameterization and regional extrapolation, and suggest a wide range of possible epidemic outcomes, from terminal importations to stuttering chains of transmission to full blown epidemics [4, 9–11]. Once an outbreak is underway, early case data can be used to directly estimate *R*_0_ [12–14]. For arboviruses with environmentally constrained spatial heterogeneity, such case-based estimates cannot be easily extrapolated from one region to others.

Here, we introduce a method for performing real-time updating of ecologically informed estimates of *R*_0_ that reconciles discrepancies between initial estimates of *R*_0_ and real-time data on local transmission of an emerging arbovirus. This approach is applicable to settings in which there is an ongoing threat of arbovirus importation and in which transmission has not precipitated a large-scale epidemic. In particular, this work was motivated by recent introductions of ZIKV into the continental US. As hundreds of cases arrived from affected regions throughout the Americas, officials sought to estimate risks of autochthonous (local) transmission and identify high-risk regions in the southern US. However, given the novelty of ZIKV and the large proportion of ZIKV cases that go undetected, early ecological estimates had high uncertainty [5, 6, 15–17]. Our method combines indirect and direct estimation methods to reduce such uncertainty and increase accuracy at high spatiotemporal resolution. We first build prior ecological estimates of local *R*_0_ and then harness real-time importation data—cases that arrive in a naive location with or without subsequently infecting others—to update the estimates, while explicitly modeling case reporting uncertainty. As a case study, we use the almost complete absence of secondary transmission following 298 importations of ZIKV into the state of Texas in 2016 and 2017 to lower and narrow local estimates of *R*_0_.

## Materials and methods

We used a two-step procedure to estimate the monthly *R*_0_ for each of the 254 Texas counties (hereafter county-month *R*_0_): (1) estimate *a priori* county-month *R*_0_ distributions using published ecological models of ZIKV transmission [4, 6], and (2) using these as Bayesian priors, generate posterior *R*_0_ distributions based on reported importations and subsequent local transmission.

### Data

We analyzed all known ZIKV importations into Texas from January 2016 to September 2017, including the county and notification date; county-level purchasing power parity (PPP) in US dollars [18]; daily temperature data at a 5 km x 5 km resolution for 2016-2017 and historical averages from 1960-1990 [19, 20]. For each county and month, we averaged daily temperatures across all 5 km x 5 km grid cells whose center fell within the county; we aggregated 5 km x 5 km mosquito (*Aedes aegypti*) occurrence probabilities similarly [21]. Those data are available at dx.doi.org/10.18738/T8/HYZ53B.

In all, six mosquito-borne, autochthonous cases of ZIKV were reported in Texas in 2016 and one was reported in 2017 [22]. For updating *R*_0_ estimates, we analyzed 2016 data where two autochthonous cases were detected in Cameron County from passive surveillance–one in November and one in December 2016; we excluded four nearby cases discovered during the November follow-up investigation, because our model does not incorporate active surveillance. As sensitivity analyses, we re-estimated *R*_0_ assuming that no cases were detected and that all six cases were detected (S1 Fig). Accounting for this worst-case scenario lead to increased estimates for risk across the state, but expected risk still remained well below epidemic potential. For validating our estimates, we analyzed 2017 data and considered only one of the two reported autochthonous cases, as the second case occurred outside the timeline of our 2017 importation data.

### A priori county-month *R*_0_ estimates

For ZIKV to be locally transmitted in a location, a mosquito must bite an individual, the mosquito must be infectious with the virus, and then that mosquito must bite a susceptible individual and transmit that virus. We focus our analysis on a singular value, *R*_0_, which captures the individual contributions of these factors on the potential for human to human transmission. Following Perkins *et al*. [6], we estimated *R*_0_ using a temperature-dependent Ross-Macdonald formulation,

$$\begin{array}{c} R_{0}\left(T\right)=\frac{mbca^{2}e^{-\mu (T)n(T)}}{\mu (T)r}, \end{array}$$ (1)

with parameters as defined in Table 1. Although others have considered temperature dependence in parameters other than *μ* and *n* (e.g., [23, 24]), we opted for our relatively simple formulation for a few reasons. First, effects of temperature on *μ* and *n* are likely to be the most critical to include, due to their relatively strong influence on *R*_0_ [25]. Second, whereas temperature can influence *m* in multiple ways [23], we elected to model *m* in an alternative fashion that incorporates influences from a wider range of factors than temperature alone. Third, our approach for scaling *R*_0_ through data assimilation—which is the primary goal of this work—is applicable to essentially any formulation of *a priori* county-month *R*_0_. The *R*_0_ prior used here is particularly germane to the real-time nature of this exercise, given that an early (but largely similar) version of it was available as a preprint near the very beginning of the time window of our analysis [26].

**Table 1:** Parameters used to create prior *R*_0_ estimates from the literature.

Parameter	Description	Distribution	Value (CI)	Source
*b*	Mosquito-to-human transmission probability	Constant	0.4	[27]
*c*/*r*	Human-to-mosquito transmission probability times the duration of human infectiousness (days)	Constant	3.5	[28]
*a*	Mosquito biting rate	Constant	0.67	[29]
*μ*(*T*)	Mosquito daily mortality rate	Generalized additive model	0.00002 (0.000008, 0.287)^1^	[25, 30]
*n*(*T*)	Extrinsic incubation period in mosquitoes (days)	Exponential	6.1 (3.4, 9.9)^1^	[31]
*m*	Mosquito-human ratio	Spatially variable with *Ae. aegypti* and G-Econ indices	0.36 (0.20, 0.60)	[6]

^1^. At 30 °C

The ratio of mosquitoes to people, *m*, makes use of two spatially varying inputs: *Ae. aegypti* occurrence probability and an index of economic purchasing power. The first of these was based on a global collection of *Ae. aegypti* occurrence records used to inform an occurrence probability model that made use of a number of environmental variables, including a temperature suitability index, precipitation, enhanced vegetation index, and urbanicity [21]. The second of these features spatial estimates of purchasing power parity (PPP) [18], which offers a relative spatial index of economic wealth that other work [6, 32] has shown can serve as a useful proxy for spatial variation in economic factors affecting mosquito-human contact. Here, we used gridded estimates of those two spatially variable inputs for Texas, but otherwise followed the methodology of Perkins *et al*. [6] for translating them into a gridded surface of *m*. In brief, this involved transforming mosquito occurrence probability into a proxy for mosquito abundance and multiplying it by a shape-constrained additive model of PPP, which resulted in a convex, monotonically decreasing relationship between PPP and *m*. We do not consider the near-term effects of precipitation on local ZIKV transmission risk, but one could be included in future iterations, given a validated *a priori* model describing the relationship.

To derive *a priori* distributions of *R*_0_ for each county-month, we drew 1,000 Monte Carlo samples from each parameter underlying the *R*_0_ formulation described above. This accounted for uncertainty in *μ*, *n*, *Ae. aegypti* occurrence probability, and the relationship between PPP and *m*, consistent with previous descriptions of uncertainty for each of those parameters [6]. These parameter draws were applied to the appropriate county and month data for Texas. Finally, we fit gamma distributions to each probability distribution for local transmission risk for use as informative priors, see S2 Fig for comparisons between the samples and the fitted gamma distributions.

### Autochthonous transmission likelihood

We developed a likelihood function describing the expected outbreak size following an importation, using the approach from [13]. Assuming that the secondary case distribution for each infected individual is negative binomial with mean *R*_0_ and dispersion parameter, *k*, and that all cases are detected, then the probability of an outbreak of chain size, *j*, is can be described by [13, 14] as

$$\begin{array}{c} s\left(j,R_{0},k\right)=\frac{\Gamma (kj+j-1)}{\Gamma (kj)\Gamma (j+1)}\frac{{\left(R_{0}/k\right)}^{j-1}}{{\left(1+\left(R_{0}/k\right)\right)}^{kj+j-1}} \end{array}$$ (2)

where Γ(*n*) = (*n* − 1)!. However, not all cases are detected and we assume the imported index case is always detected and correctly classified as an importation, so the probability of detecting a chain of size, *j*, from a given importation is given by

s*(j,R0,k,pd)=∑l=j∞s(l,R0,k)·(l−1j−1)·pdj−1·(1−pd)l−j (3)

where *p*_*d*_ is the case detection probability [13, 14]. This equation can be understood intuitively. If cases can be missed then there are infinitely many ways in which an outbreak of a certain size can be detected (e.g. 5 detected cases could stem from an outbreak of size 6 with 1 case missed, or size 7 with 2 missed etc). This equation enumerates those possibilities and sums their individual marginal probabilities. Importantly, this allows for local, undetected cases. We take the product of all likelihoods for each imported case as

$$\begin{array}{c} L\left(\vec{O}|\alpha ,\vec{R_{0}},k,p_{d}\right)=\prod_{i=1}^{\text{length}(\vec{O})}s^{*}\left(O_{i},\alpha {R_{0}}_{\gamma_{i},\omega_{i}},k,p_{d}\right) \end{array}$$ (4)

where $\vec{O}$, contains the observed outbreak sizes for each importation (terminal importations have an outbreak size of one), ${R_{0}}_{\gamma_{i},\omega_{i}}$ denotes the county(*γ*)-month(*ω*) *R*_0_ for the location and time that the importation occurred, and *α* is a statewide scaling factor applied to each ${R_{0}}_{\gamma_{i},\omega_{i}}$. The introduction of the state-wide scaling factor allows for localized importations to inform statewide estimates, but assumes that biases in the *a priori* *R*_0_ estimation procedure are constant across counties and months. While our analysis does not assume that all imported cases are detected, we do not explicitly model missed imported cases and simply explore the consequences of these potentially missed cases in a sensitivity analysis. If systematic biases in the spatiotemporal detection of imported cases are discovered, our method should be updated accordingly. Details of simulations and validation of the likelihood can be found in S1 File and S3 Fig.

### Estimating the dispersion parameter

The negative binomial dispersion parameter governs the variability in secondary cases following each importation, with values near zero meaning that most importations yield few or no cases while a few “superspreaders” produce many. Superspreading dynamics are known to occur with mosquito-borne diseases like ZIKV [33, 34]. We assume that ZIKV secondary case distributions are the same as that of dengue virus (DENV) [35], though these estimates should be updated as ZIKV-specific secondary case distributions are identified. Padmanabha et al. describe the relationship between regional *R*_0_ and the percentage of DENV imported cases that generate over 20 secondary infections (*p*_20_), as *R*_0_ = 0.63 × 100 × *p*_20_ + 0.58. As we require *p*_20_ > 0 for all values of *R*_0_, we set *p*_20_ to an arbitrarily low, but non-zero value (*p*_20_ = 1 × 10^−8^) for values of *R*_0_ < 0.58. We then found the dispersion parameter for every value of *R*_0_ that lead to the expected *p*_20_ value from that previously estimated relationship. The result was that a single dispersion parameter captured the relationship well for all *R*_0_ values and thus we set the dispersion parameter, *k* = 0.12, for all analyses (S4 Fig). The highly dispersed nature of the secondary case distribution suggests that most importations will not lead to epidemics, even if *R*_0_ > 1. For example, even with *R*_0_ = 1.5, we would only expect to see an epidemic stemming from ∼8% of imported cases.

### Updating posterior *R*_0_ estimates

Given our set of prior distributions for county-month transmission risk, we set out to estimate the statewide scaling factor, *α*, as a means to update our *a priori* transmission risk estimates. We estimate the posterior distribution for this scaling factor for each day with a new importation between January 2016 and January 2017 (our method also explicitly updates county-month *R*_0_ estimates for county-month combinations with imported cases). Importantly, this doesn’t indicate that *α* is changing through time, but rather that we refine our estimate for it as more data accumulate. We assumed a uniform prior for *α* of *U* ∼ (0, 2), and used a blocked Gibbs sampling algorithm of MCMC. For each MCMC step we provide the detected imported cases to date and propose each county-month *R*_0_, a single *α*, and a probability of case detection, *p*_*d*_. County-month *R*_0_ proposals were normally distributed around the previous sample with standard deviation of 0.1, *α* proposals were distributed *U* ∼ (0, 2). We used a previously estimated distribution for *p*_*d*_ as a strong, informative prior, *p*_*d*_ ∼ *N*(5.74%, *sd* = 1.49%), as there are identifiability issues given its relationship with *α*, and assumed it to not vary spatiotemporally [32]. There were no differences between the posterior and prior distributions for the reporting rate S5 Fig. We used the Metropolis-Hastings probability to accept or reject proposals. Our chains consisted of 200,000 samples with a burn-in duration of 100,000, thinning every 10 steps. We ran a single chain for each parameter set, and assessed convergence through visual inspection of the trace plots (S6 Fig). Further algorithmic details and code are available on Github (https://github.com/sjfox/rnot_updater). To help with an intuitive understanding of the method we also present a hypothetical situation (S1 File and S7 Fig).

### Validating posterior county-month *R*_0_ estimates

We derived the expected number of autochthonous cases from the importation data through September 2017 (at that time, the most recent importation was detected in mid-May) and compared the estimates to the actual reported autochthonous cases. We incorporated uncertainty into our estimates by sampling from the posterior county-month *R*_0_ distributions and simulating outbreaks accordingly (full details in S1 File).

## Results

In the face of a novel emerging infectious disease threat, public health officials must borrow information from related diseases and/or from epidemics in geographically distinct regions. Adapting these models to new regions necessarily increases the uncertainty in risk estimates, but can prove invaluable for data-driven public health decision-making. Here we present a rational framework for updating these initially uncertain estimates with real-time case data from the emergence of Zika in Texas. The results are structured as such: we first present data from Texas’ baseline importation and transmission risk estimates, we then update those estimates with importation and local transmission data, and finally present findings from validating those estimates using out of sample data.

### Baseline importation and transmission risks in Texas

**Fig 1:**
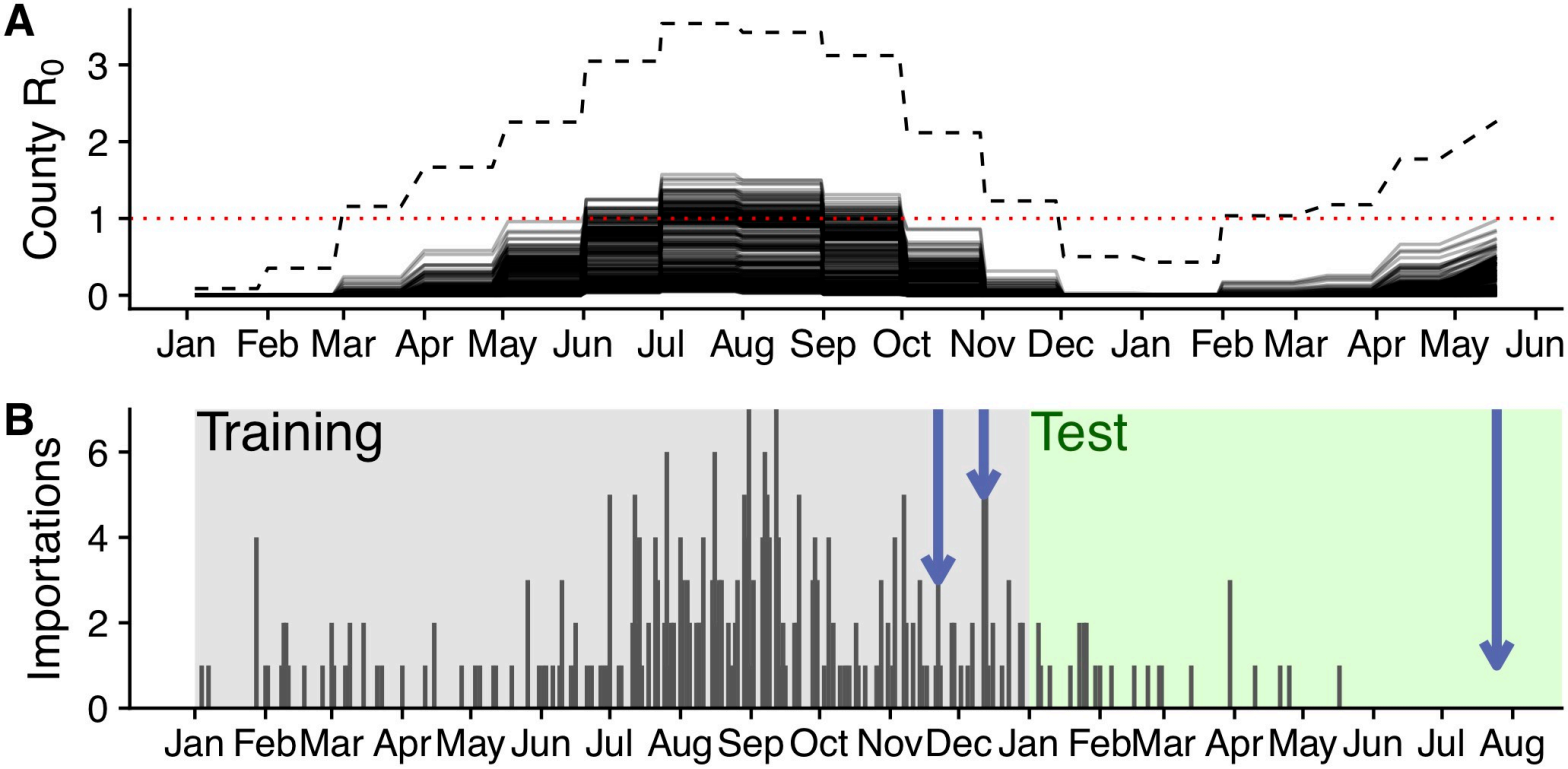
Texas importations and baseline transmission risk estimates for 2016-17. (A) Initial ZIKV *R*_0_ estimates using ecological risk models parameterized with actual 2016-2017 temperatures. Each solid line shows median values for one of Texas’ 254 counties. Dashed line shows the highest upper bound (99th percentile) across all counties. Horizontal dotted red line illustrates the threshold for county-month epidemic risk (*R*_0_ = 1). (B) Daily ZIKV importations into Texas. Blue arrows indicate importations that produced detected autochthonous transmission; shading indicates training (2016) and testing (2017) periods.

Prior to making importation-based updates, our initial median estimates of *R*_0_ across Texas’ 254 counties in 2016 range from approximately 0 to 1.5 throughout the year with July and August having the highest transmission risk (Fig 1A). Given the potential implications for determining the epidemic potential of a county, throughout the manuscript, we conduct a one-sided test at a 1% significance level of the resultant probability distributions. We thus take a conservative approach and only consider counties whose probability distribution’s 99th percentile (upper bound) is above one to be at risk for an epidemic (*R*_0_ > 1). Simply put, we only classify counties as no epidemic risk if there is less than a 1% chance of *R*_0_ > 1. Initial upper bound estimates reach as high as three, and 119 (47%) of Texas counties are expected to be at risk of a local epidemic in at least one month of the year (Fig 1A, S8 Fig). When we considered historic average temperatures rather than 2016 temperatures, the projected 2017 risks were consistently lower, with the largest differences occurring during the unseasonably warm 2017 winter (S9 Fig). Case importations peaked in July, August, and September of 2016, with 164 (55%) of the 298 total 2016 importations arriving then (Fig 1B). The few detected autochthonous cases occurred in November and December, when expected risk was relatively low but not negligible.

### Updated transmission risks in Texas

**Fig 2:**
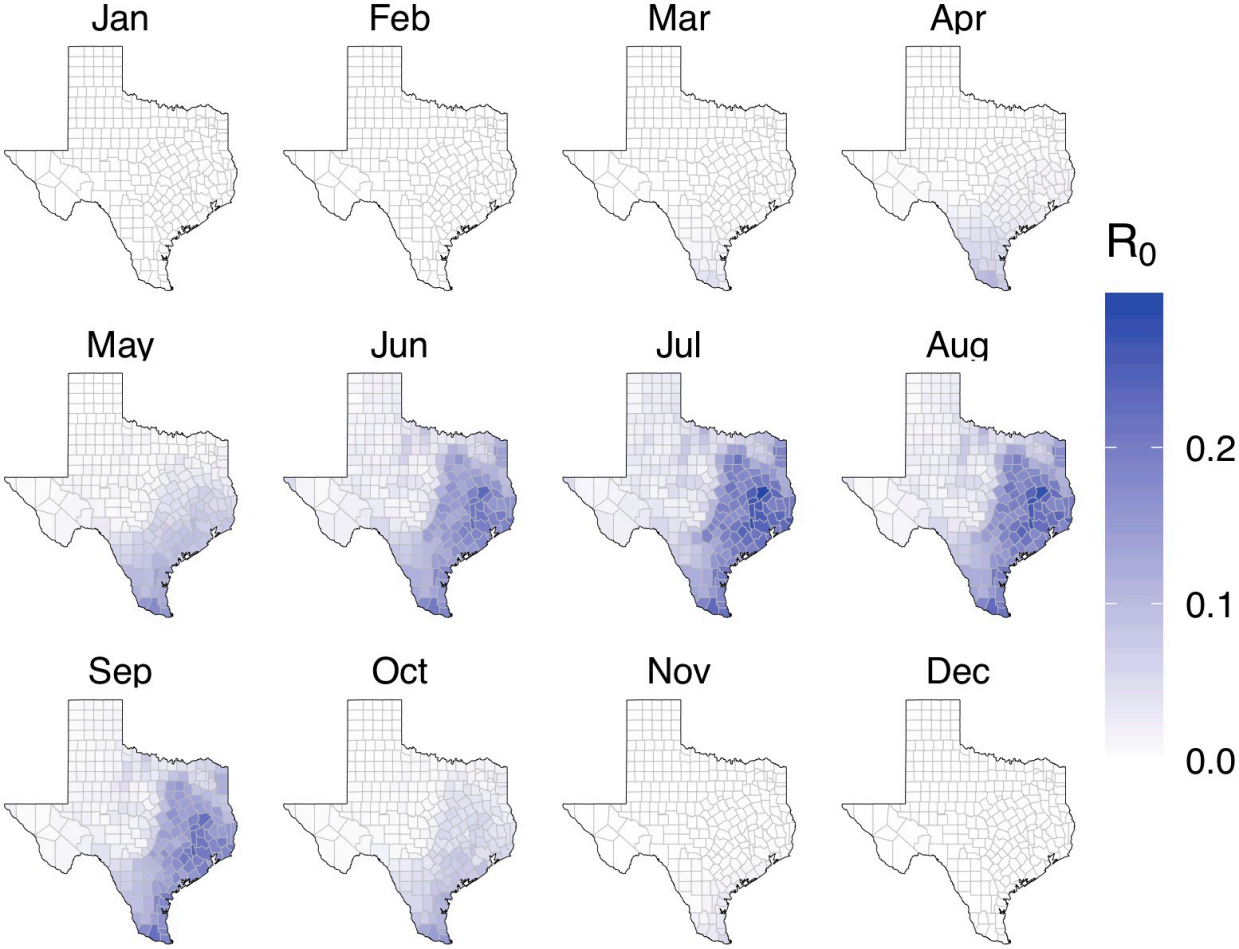
Posterior median county *R*_0_ estimates for Texas, based on ZIKV importations through January 2017. This assumes that all importations were terminal except for two autochthonous cases detected in Cameron County in late 2016.

Based on all importations and autochthonous cases that occurred in Texas prior to January 2017, we estimate that all Texas counties have a median posterior *R*_0_ below 1 (Fig 2). Median estimates range from 0 to 0.29; upper-bound estimates range from 0 to 1.12, with only six (5%) of the original 119 high-risk counties maintaining epidemic potential (S10 Fig). The six counties with remaining epidemic risk are all contained within the greater Houston metropolitan area, and all have relatively low estimated transmission risk estimates ranging from 0.25-0.29 (Grimes, Houston, Madison, Montgomery, Walker, and Waller counties). When we assume historic averages rather than 2016 temperatures, we obtain similar results (S11 Fig).

In a sensitivity analysis that assumes ∼20 times more undetected importations, we found that the estimated risks decreased further (S1 Fig). We also varied the number of detected autochthonous cases in November: as they decrease from one to zero, the estimated risks decrease considerably; as they increase to five, estimated risks increase, with 83 counties becoming at risk for a local outbreak (S1 Fig). However expected transmission risk for these counties is still well below 1, ranging from 0.3-0.6.

**Fig 3:**
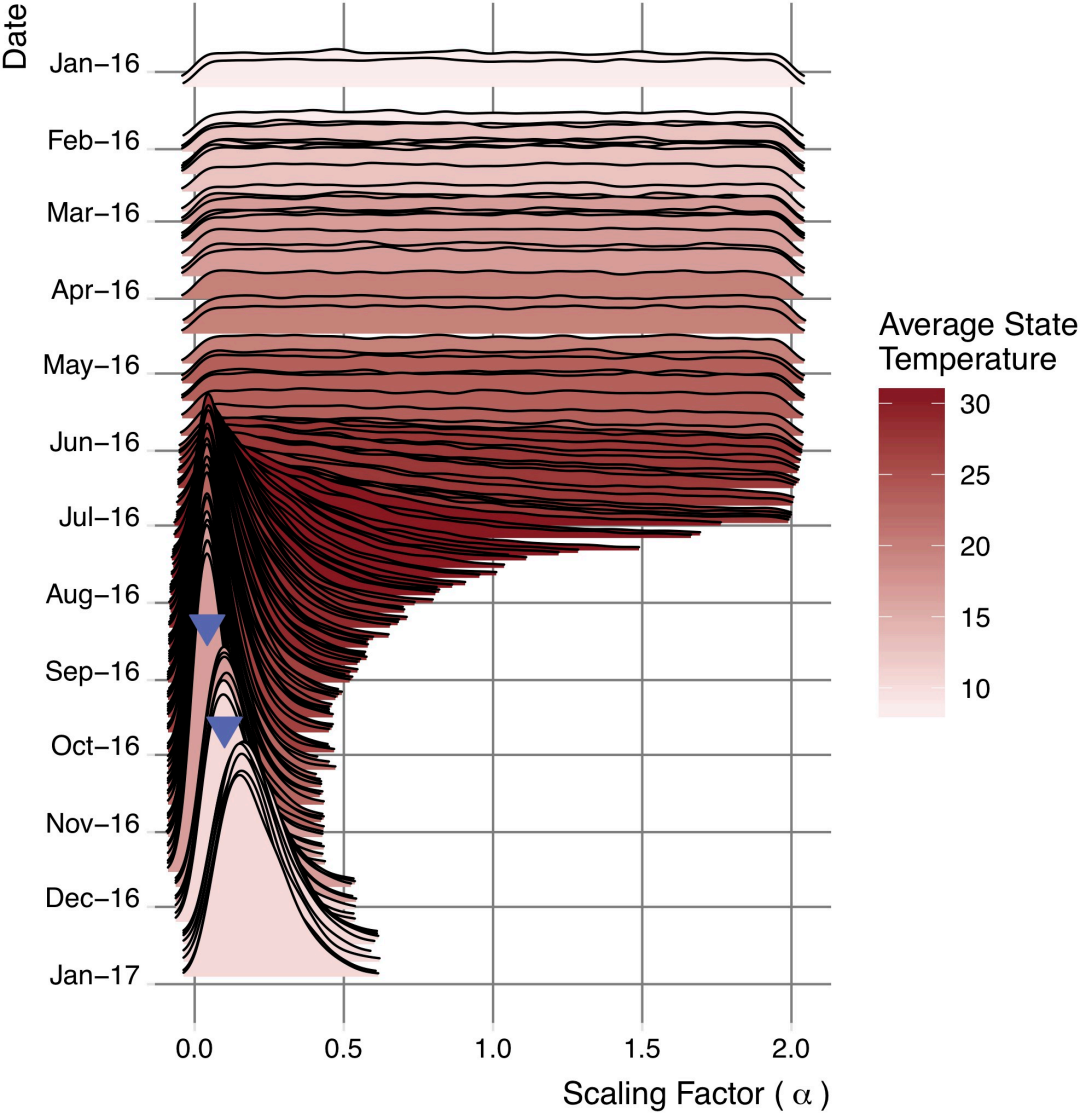
Evolving posterior distribution of statewide scaling factor for *R*_0_. Zika importations, both with and without subsequent detected autochthonous transmission, provide insight into local transmission potential, via a statewide scaling factor, *α*. This shows the posterior distributions of *α*, for each day of 2016 that had at least one imported case. Median estimates reach a minimum in early November, just before the detected autochthonous transmission events (upside-down blue triangles). Red shading indicates the average statewide monthly temperature. Note: the scaling factor is never less than zero.

Importation events had variable impacts on the posterior estimates, depending on their timing and location (Fig 3). Terminal importations early in the year, when *a priori* *R*_0_ estimates were low, had little effect; those arriving in the summer months, when high *a priori* *R*_0_ estimates suggested that transmission should have occurred, led to sharp decreases and a shrinking confidence interval. By early November, the median *α* decreased from 1.0 to 0.06 with a 95% CI of 0.002-0.30. The two secondary transmission events detected in November and December increased the posterior *R*_0_ estimates and widen the confidence interval slightly, but did not provide enough evidence to qualitatively change the transmission risk estimates. Incorporating all data up to January 2017, our best estimate is that *R*_0_ values across the state are roughly one fifth the original estimates (median: 0.19, 95% CI: 0.05-0.48).

Interestingly, based on the *a priori* *R*_0_ estimates, we would have predicted local transmission to occur during the summer when importations and transmission risk were high. In fact, these initial expectations for transmission were lowered even further prior to the detected locally acquired case in November. Since we estimated the posterior distributions for transmission risk and the statewide scaling factor jointly, we were able to compare the prior and posterior estimates to understand if specific counties were downgraded more or less than expected by the statewide average. We find that the ratio between prior and posterior estimates tends to average near the statewide scaling factor of 0.19, except for counties that experienced many reported importations during the year (S12 Fig). These counties tended to have lower than average ratios like Harris, Dallas, Tarrant, Bexar, and Cameron counties (S12 Fig). While the timing of the autochthonous transmission may not have been predicted using our initial risk estimates, the transmission events both occurred in the southern tip of Texas (Cameron county), which was predicted to be the highest risk county during those months.

The temporal mismatch between the model predictions and the observed ZIKV local transmission in November and December might stem from the a priori transmission risk model, which constrains posterior estimates. Specifically, the model does not account for potential time lags between ZIKV environmental suitability and eventual emergence of locally acquired cases. Environmental determinants of mosquito population growth may take several weeks or months to impact arbovirus risk. Furthermore, the timing of case reports following infection will depend on the timing and severity of symptoms, healthcare seeking behavior and clinical reporting. To assess how this effect might alter results, we incorporated a two month lag in our *a priori* suitability model as estimated in [36]. In essence we shifted transmission risk estimates two months later than our baseline predictions suggested. For example importations occurring in November are matched with baseline transmission risk estimates from September. The resulting model yielded estimates that are more consistent with the observed ZIKV local transmission, although posterior risk estimates were remarkably consistent with our baseline results, with the highest risk period shifted by two months (S13 and S14 Figs). In comparing our baseline model with the lagged one, we calculate a BIC of 2.9 in favor of the lagged model, suggesting a moderate improvement to model fit.

### Expected autochthonous transmission in Texas

**Fig 4:**
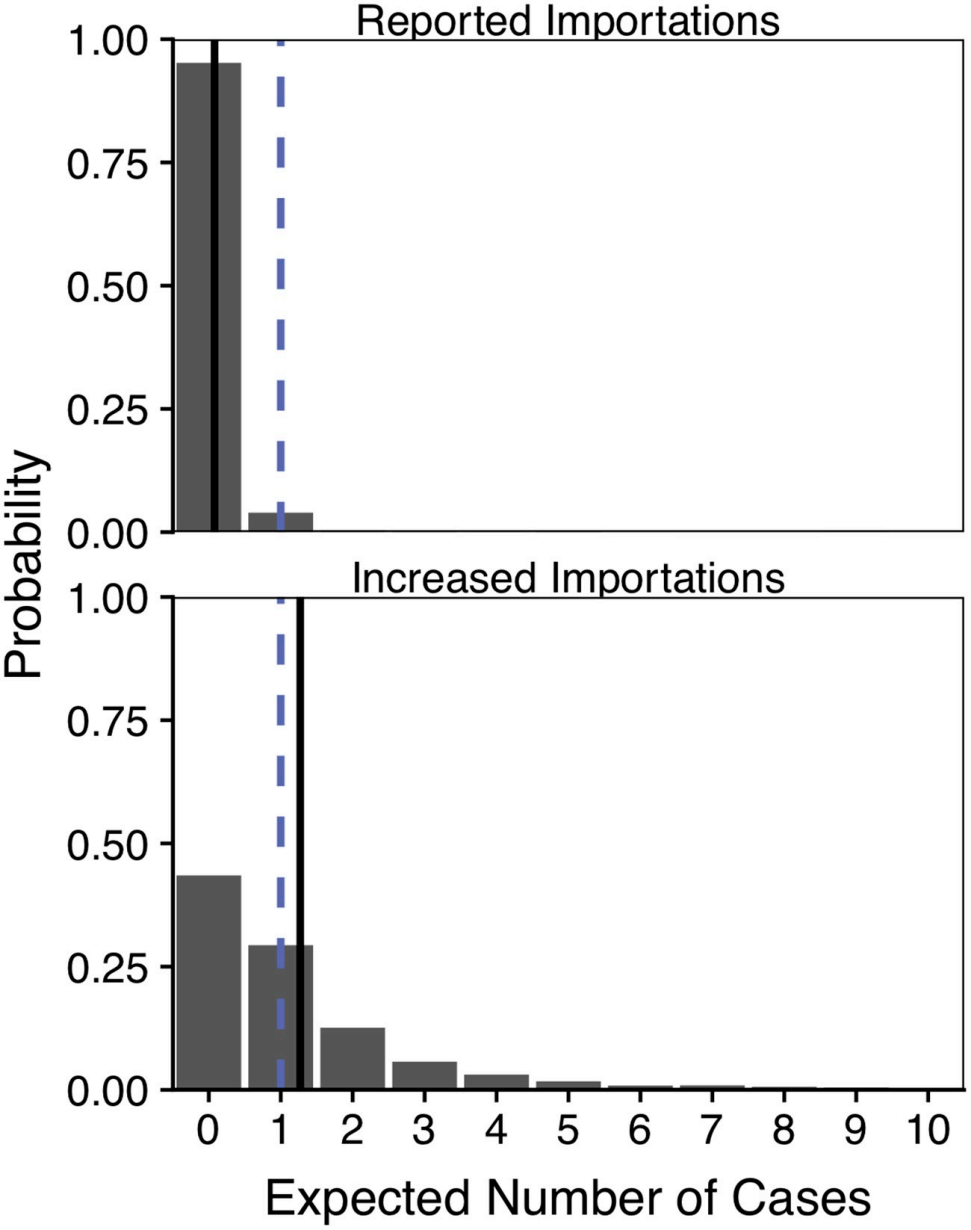
Expected autochthonous cases in 2017, assuming revised county *R*_0_ estimates through September 2017. The probability distributions are built from 10,000 simulations, each randomly drawing from the *R*_0_ posterior distributions. The dashed blue line indicates the actual number of detected autochthonous cases in state (one), and the solid black lines indicates the mean expected number of autochthonous cases for the baseline importation scenario, in which only the reported importations occurred (top) and the increased importation scenario, in which a large fraction of importations went undetected (bottom).

We use transmission risk estimates based on importations through December 2016 to estimate the number of autochthonous cases we would expect to detect in Texas in 2017. Based on our posterior estimates we estimate a 1% probability of at least one single detected case in Hidalgo county at the time the secondary transmission occurred. Since the sample size is low, we average the expected number of secondary cases over the 2017 time period where importation data were available. Assuming first that only the reported importations occurred in 2017 (26 total), we estimate that there should have been 0.08 (95%CI: 0-1) detected autochthonous cases in the state; assuming that many importations went undetected, according to the reporting probability ($\frac{26}{p_{d}}\approx 453$ cases), we estimate 1.3 (95% CI: 0-7) detected autochthonous cases. Both of these estimates are consistent with the single autochthonous case detected in Texas in 2017, though our results best fit a scenario with many undetected importations (Fig 4).

## Discussion

The global expansion of ZIKV was declared a Public Health Emergency of International Concern in February 2016, and caused more than 565,000 confirmed or probable cases and over 3,352 documented cases of congenital Zika syndrome. Although it is receding in most regions of the world, ecological risk assessments suggest that previously unaffected or minimally affected areas may remain at risk for future emergence, including parts of Asia and South America [37–39]. Differentiating regions that can sustain a ZIKV epidemic (*R*_0_ > 1) from those that cannot is vital to effective planning and resource allocation for future preparedness plans. To address this challenge, we have developed a simple method for refining uncertain risk assessments with readily available data on disease importations.

Using previously validated ecological suitability estimates as a starting point, we applied the method to update ZIKV *R*_0_ estimates for each of the 254 counties in Texas, and found that only six counties have non-negligible probabilities of sustained local transmission, though an additional 77 counties may also be at low risk if our assumptions about local case data are too liberal. This is a substantial downgrade in expected risk, given that 43% of the 254 counties were previously thought to be vulnerable to ZIKV outbreaks, and even counties still at risk for epidemics on average had *R*_0_ estimates downgraded by 80% [4]. Based on these estimates and the number of importations that occurred in the state, our refined model suggests that there should have been between zero and one detected case of locally acquired ZIKV between January and September of 2017, which corresponds to the single transmission event actually detected in Cameron County in July 2017. Interestingly, a model that incorporates the possibility of many more missed imported cases into the state of Texas fits the data better, with an expected number of cases close to one (Fig 4).

There is a discrepancy between our model predictions and the ZIKV case data: our final model predicts the highest local transmission risk to be in the summer across the state, but most cases of local ZIKV transmission occurred in the winter (6 out of 7). While these case numbers are low and may simply reflect random variation, the most recent dengue outbreak in South Texas peaked in November, concurrent with a large dengue epidemic in Northern Mexico, suggesting that fall may be a higher risk season for arbovirus transmission than our final predictions indicate [40]. The discrepancy likely stem from errors in our initial risk estimates, which serve as informative priors for our analysis. Our model does not incorporate a time lag between case importation, local transmission, and case detection, which has been previously estimated around two months [23, 36, 37]. Incorporating a two month lag in our model slightly increased the fit of the resulting predictions (BIC = 2.9), but does not produce qualitatively different results in the magnitude of county risk other than the temporal shift S14 Fig. While these results were promising, we chose to present our baseline results instead of those from the lagged model, because the baseline model has been previously validated [6]. Importantly our simplistic lagging procedure does not properly encapsulate the mechanistic intricacies determining the relationship between environmental suitability, mosquito population growth, case importation, local transmission, and subsequent detection that account for the lags outlined in [36]. Our results support these time lags as a crucial element for predicting ZIKV risk, and highlight the need for future research into the mechanistic underpinnings to reduce bias and improve model fit and predictive accuracy even further.

The factors driving county transmission risk were primarily temperature and mosquito population numbers (occurrence probability). Remaining uncertainty in county risk stems from a lack of detected imported cases, as imported cases primarily helped to downgrade Zika risk in the absence of local transmission. This provides the counterintuitive explanation for why our model suggests low epidemic risk for Harris county (containing the city of Houston) even though it was by far the county with the most imported cases. Given comparable importation and local case data, this approach could readily update ZIKV transmission risk estimates for any county in the continental US.

Our estimation method relies on several simplifying assumptions, which we divide into those concerning this ZIKV test case of the updating procedure, and those concerning the updating procedure itself. For this specific implementation, we assumed that the shape of the secondary case distribution resembles that of dengue. Although we have no evidence to the contrary, this should be updated as ZIKV-specific estimates become available [35]. We also assumed that transmission is equally likely from imported and locally acquired cases. Imported cases may be less infectious than locally acquired cases for two reasons, leading us to underestimate local transmission risks. First, they may be more likely to receive care that limits transmission, although most ZIKV cases are inapparent or mild and do not require medical care [16], and second their local infectious periods may be shorter than those of autochthonous cases. This assumption could be relaxed in cases where data are available on the relative detection rates. In this case, we also do not consider the possibility of sexual transmission of ZIKV. While sexual transmission has occurred and may be important for specific populations [41], we assumed that mosquito-borne transmission is the dominant mode of infection. Our current iteration of the model makes several simplifying assumptions related to how we handle the temporal dynamics of both the *a priori* risk estimation and the updating procedure. Our baseline transmission risk model does not take any outside events that could modulate future risk into consideration (e.g. rainfall in the previous month causing upticks in mosquito populations), and future model development will be necessary to account for these important temporal dynamics [23, 36, 37]. For example, approaches that account for variability in the serial interval between successive cases and distributed lagged effects of environmental variables on transmission potential may hold promise for addressing this limitation [42].

The updating method itself also requires several simplifying assumptions. First, it relies on informative Bayesian priors for the relative spatiotemporal risk of a region. Posterior estimates could be biased by errors in these priors. At this point, we lack sufficient data from Texas to improve our estimates of the component parameters of *a priori* *R*_0_. The lack of such data, in fact, motivated our more modest goal of refining estimates of relative risk across counties. We could potentially improve model accuracy by incorporating smoothing techniques that more realistically capture spatiotemporal correlations and heterogeneity in transmission risk across Texas. This method also treats all importations as independent. However, spatiotemporal heterogeneity in case detection probabilities or clustering of cases (e.g., testing of travel companions) could bias risk estimates. When secondary clusters are detected, we assume they share a transmission tree stemming from a single detected importation. In this case, the low ZIKV detection rate suggests that both primary importations and secondary cases are likely to be missed. If the detection rates between the two types of cases are roughly similar our results hold. If importations are detected at higher rates than secondary cases, then the resulting risk estimates will be higher; when we assume the reverse, meaning locally transmitted cases are more likely to be detected than imported cases, the posterior risk estimates will be reduced. The additional assumption, that clusters are epidemiologically connected, seems reasonable for the small contained outbreaks detected in Texas, but may not be appropriate for importation-fueled arbovirus outbreaks in Florida, for example. In such cases, molecular data might enable estimation of transmission clusters [43, 44]. Furthermore, there are identifiability issues associated with estimating the statewide scaling factor alongside the case detection probability, as both parameters modulate the expected number of secondary cases in the same fashion. We therefore used a strong, informative prior for the case detection probability from a previous study, and found no changes in the posterior distribution S5 Fig. If case detection is lower than we assumed, then the detected cases in Texas would have had to arise from larger outbreaks, so our final risk estimates would be closer to the prior (larger). Alternatively, if case detection in Texas is higher than we assumed, there would be an even larger discrepancy between our prior and resultant posterior risk estimates S15 Fig.

During the height of the ZIKV threat, public health agencies in the US rapidly implemented both preventative measures (e.g., vector control and educational campaigns) and response measures (e.g., laboratory testing and epidemic trigger plans), particularly in high-risk southern states. Decision makers sought to identify and narrow the spatiotemporal scope of outbreak risk to enable targeted responses, efficiently allocate resources, and avoid false alarms [15, 45]. Our method facilitates such rapid, real-time geographic risk estimation from typical early outbreak data, and allows for real-time updating of estimates as new data arise. In interpreting our results from 2016 in retrospect, they would suggest that the baseline transmission risk combined with the public health response was sufficient to mitigate the threat of a ZIKV epidemic across the state. Our method cannot disentangle the impact of public health response from the underlying transmission risk, so we cannot estimate the impact of the public health interventions. In real time, our results would have validated the magnitude of the public health response through the summer of 2016, and likely alleviated some of the epidemic concerns that arose in the winter when a handful of locally transmitted cases were detected in the state.

Critically, we can conclude neither that all initial ecological risk assessments for ZIKV will overestimate risk, although this seems to be the case for ZIKV in Texas and elsewhere in the US [9], nor that public health preparations and interventions for ZIKV are no longer necessary in Texas or the southern US. Rather, our results suggest that sustained ZIKV outbreaks are unlikely, but not impossible (3% of the state remains at low epidemic risk), and provide more robust and localized estimates of ZIKV risk that can inform more targeted surveillance and reactions to future ZIKV importations. While our method provides an avenue to improve imperfect *a priori* risk estimates using real-time data, future predictions and estimates could be improved with advances to our mechanistic understanding of transmission risk (e.g., incorporating rainfall or improved understanding of mosquito-human contact patterns) [2]. These estimates should also only be taken as the baseline level of risk for the state. Climate change, anomalous weather events, or unforeseen circumstances that alter mosquito populations or mosquito-human interactions could raise or lower the Zika risk in Texas [3, 46].

This framework is novel in its integration of *a priori* ecological transmission risk estimates with updating directly from real-time case reports [13, 14]. It thereby provides increasingly accurate and precise risk assessments to support public health decision making, and can be generalized to update *R*_0_ estimates from importation data, regardless of the *a priori* method of estimation. For example, a new approach combining epidemiological and molecular analyses suggests that transmission risk in Florida is subcritical (i.e., *R*_0_ < 1) [44, 47]. Given that Florida experienced thousands of introductions, only a few of which sparked large outbreaks, coupling such outbreak-driven estimation with our terminal importation method may provide a powerful real-time risk assessment framework for exploiting all available data. This method resembles those used to assess disease transmission risk during elimination efforts, including malaria in non-endemic regions [48]. The key innovation is that, by starting with ecological suitability maps, we simultaneously identify important transmission hotspots and leverage case data from one region to inform risk estimates elsewhere.

We presented a simple and rational method for continuously updating transmission risk estimates for populations experiencing infectious disease importations, with or without secondary transmission. As we demonstrated for ZIKV in Texas, large numbers of terminal importations can profoundly lower both estimated risks of transmission and uncertainty in prior estimates, particularly those derived from ecological suitability or other models that borrow inputs from related pathogens in other parts of the world. Expanding our model to other regions could sharpen transmission risk estimates, and allow for more targeted public health interventions in the remaining hot spot locations [5, 9, 17]. Transmission risk estimates always include uncertainty, but by assimilating data in real-time, our method can help confirm or revise public health understanding in the midst of an outbreak. Although the threat of ZIKV emergence in the continental US motivated this study, this new framework can be applied to improve transmission risk assessments when a disease newly threatens a population via regular introductions with minimal secondary transmission.

## Supporting information

S1 File Supplemental information. (PDF)

S1 Fig Sensitivity analysis of posterior *R*_0_ estimates for each county. Each point indicates a county-month posterior *R*_0_ estimate under different estimation scenarios. The x-axis value for all points is determined by the baseline scenario where posterior *R*_0_ estimates consider only a single case detected in the November. The y-axis value is based on three sensitivity scenarios: (1) posterior estimates assuming no secondary transmission (dark grey), (2) five cases of secondary transmission in November (black), or (3) a single case of secondary transmission, but increased overall importations (light grey). Points falling above the black dashed line indicate that that a given scenario increases posterior *R*_0_ estimates compared to baseline estimates, and points below the line indicate the opposite. Estimates are compared for the median (left), and the 99th percentile (right) of the county-month distributions. Posterior *R*_0_ estimates increase if more secondary transmission is assumed, and decrease if less secondary transmission occurs, or if the absolute number of importations is increased.
(TIFF)

S2 Fig Comparison between the empirical prior transmission risk distributions (points) and the fitted gamma distribution for three example county-month combinations (lines). (TIFF)

S3 Fig Comparison between analytical likelihood predictions and simulations. We compare the probability mass functions for the outbreak sizes for our simulations (bars) with the analytical expectation (red dots). Rows demonstrate four different transmission risk scenarios (*R*_0_), and columns describe three different scenarios: (1) where every case within a transmission chain is detected (Perfect), (2) where all cases are detected independently with a specific reporting rate (Imperfect), and (3) where all cases are detected independently with a specific reporting rate except for the index case which is always detected (Imperfect Import). The Imperfect Import probability mass function is the one used for all analyses in this article. All simulations are completed with a reporting probability of 0.0574, and *k* = 0.12.
(TIFF)

S4 Fig Match between estimated and assumed dispersion parameter. Probability of a single importation generating 20 secondary infections from (29) (Line), or using our assumed dispersion parameter and negative binomial distribution (Points).
(TIFF)

S5 Fig Comparison between the informative reporting rate prior distribution (Red line) and the posterior distribution (histogram). (TIFF)

S6 Fig Trace plots used for assessing MCMC convergence. The plots detail the posterior distribution for alpha assuming the actual temperature from 2016 and 2017, and either 0, 1, or 5 detected cases in the year.
(TIFF)

S7 Fig *R*_0_ updating using importation data. Consider a hypothetical scenario in which the first 15 terminal ZIKV importations into Texas arrive in Harris county (which includes Houston) during August 2016. (A) Estimated Harris county *R*_0_ for August 2016 *a priori* (dark grey) and after accounting for the 15 (light grey) terminal importations (Future August). These distributions are composed of 1,000 samples from the prior and posterior distributions (respectively). (B) Median *R*_0_ estimates for August before (August 2016) and following (Future August) the importation-based update.
(TIFF)

S8 Fig Prior *R*_0_ estimates for Texas counties for each month. Median and 99 percentiles are shown for each County. Fill color indicates the estimated Median or 99 percentile estimate for that county for the given month, with counties showing yellow or red indicating their *R*_0_ is above one (labels). Estimates are made for each month based on the average monthly temperature for 2016.
(TIFF)

S9 Fig Comparison between prior *R*_0_ estimates using the historic average temperature for months versus using the actual temperature from the months in 2016. (TIFF)

S10 Fig Posterior *R*_0_ estimates for Texas counties for each month using the actual 2016 temperatures. Fill color indicates the estimated Median or 99 percentile estimate for that county for the given month, with counties showing yellow or red indicating their *R*_0_ is above one (labels). Estimates are made using all importations through December of 2016, and assuming a single transmission event in both November and December.
(TIFF)

S11 Fig Posterior *R*_0_ estimates for Texas counties for each month using historic temperature data for running estimation procedure and priors. Fill color indicates the estimated Median or 99 percentile estimate for that county for the given month, with counties showing yellow or red indicating their *R*_0_ is above one (labels). Estimates are made using all importations through December of 2016, and assuming a single transmission event in November.
(TIFF)

S12 Fig Comparison between the prior and posterior *R*_0_ estimates as a function of the month of the year. Each point corresponds to a specific county in the state, and the colors indicate the number of importations that the county experiences during the specific month.
(TIFF)

S13 Fig Comparison between the posterior distributions of *α* from the baseline (Normal) scenario with the lagged (Lagged) scenario. (TIFF)

S14 Fig Posterior median county *R*_0_ estimates for Texas, based on ZIKV importations through January 2017. This assumes that all importations were terminal except for two autochthonous cases detected in Cameron County in late 2016, and shifts the prior transmission risk estimates by two months compared with the baseline scenario.
(TIFF)

S15 Fig Posterior estimate comparison based on changes to the the case detection probability. Dashed line indicates what the posterior estimates would be like if they matched the baseline posterior risk estimates. Colored points identify posterior estimates for different case detection probabilities. Yellow points are for a scenario where the probability for detecting cases was halved, and the purple points indicate estimates for when the probability was doubled.
(TIFF)
