# Supplementary material for: Downgrading disease transmission risk estimates using terminal importations
Source: PLoS Negl Trop Dis. 2019 Jun 14;13(6):e0007395. doi: 10.1371/journal.pntd.0007395 (PMC6594658; doi:10.1371/journal.pntd.0007395)
Supplement: S1 File — (PDF) [file pntd.0007395.s001.pdf]

Supplemental Information - Downgrading disease transmission risk estimates using terminal importations

Spencer J Fox, Steven E Bellan, T Alex Perkins, Michael A Johansson, Lauren Ancel Meyers

Outbreak Simulation

We checked our analytical calculations against simulations to ensure consistency. The outbreak simulations consist of two steps: (1) the outbreak transmission process and (2) the observation process. We conduct three simulation scenarios to test against analytical results. The first two simply confirm prior results, and the third confirms our analytical formulation. For each scenario, we obtain 10,000 simulations, and summarize the resultant outbreaks to obtain a probability mass function for the observed outbreak size. We then compare these simulated probability mass functions with the analytical calculations presented in the methods, and find close agreement (Fig S1).

1. Perfect observation. In this simulation, we assume that all cases are detected. For each simulation we begin with an initial index case. That case, and all subsequent cases, infect individuals according to a negative binomial distribution of mean, R_0, and dispersion parameter $k = 0.12$. We continue the simulation until there are no more newly infected individuals. We only focus on $R_0 < 1$ for simulation purposes, so no outbreaks grow forever.

2. Imperfect observation. In this simulation, we add the observation process to the transmission process. We therefore simulate outbreaks according to the perfect observation process described above, and then simulate the reporting process. For this, we find the total number of detected cases from the chain according to simulating a binomial detection process with probability of success equal to the reporting rate (0.0574 in this case) and total possible cases equal to the size of the chain from the transmission chain (n).

3. Imperfect Import observation. This simulation is exactly the same as the imperfect observation simulation, except for the fact that we always detect the index case. So in this case we simulate a binomial detection process with probability of success equal to the reporting rate (0.0574 in this case) and total possible cases equal to the size of the transmission chain minus one ($n - 1$).

R_0 estimate validation

To validate our posterior R_0 distributions, we used them to estimate the expected number of autochthonous cases from the importations data through

September of 2017 (at that time, the most recent importation was detected in mid-may) and compared the estimates to the actual reported autochthonous cases. We integrated uncertainty into our estimates as follows:

1. Draw a p_d from the reporting rate distribution.
2. Sum the number of importations occurring for each county-month, N,
3. Draw N, samples of the prior or posterior R_0 , distribution depending on which analysis is being conducted.
4. For each of the R_0 , values, we simulated an outbreak stemming from a single importation where each case infects individuals according to a negative binomial distribution with mean of R_0 , and dispersion parameter, $k = 0.12$. For each simulated outbreak, we simulate the detection process for the non-index cases as a binomial distribution with probability of success, p_d. We sum the detected cases for each of the N, outbreaks, to obtain, ν, the expected number of cases detected for that sample.
5. Repeat steps 1-4 10,000 times, saving ν

The distribution of ν obtained from the process described above can be compared with the true number of detected autochthonous cases from 2017 if we assume that all imported cases were reported. However, it's likely that there were a number of imported cases that were missed by surveillance. Therefore, we also analyzed a scenario with increased importations. To do so we followed the same process outlined above, except for altering step 3 to draw $N * (\frac{1}{p_d})$ samples to account for the missed cases rounding the resultant number to the nearest integer.

Importation-based updates of transmission risk

Hypothetically, suppose that the first 15 imported cases of Zika into Texas arrived in August into Harris County (which contains Houston) without any detected autochthonous transmission. Prior to these importations, environmental suitability models yielded a relatively high local risk estimate with median Harris county R_0 above the epidemic threshold of one (Fig S7A - dark grey). The lack of secondary cases following all 15 importations suggests that R_0 may be lower. Indeed, our updated estimates suggest that the Harris county R_0 is likely below one (Fig S7A - light grey). Our method leverages such county-level importation data to update R_0 estimates throughout the state (via a scaling factor), based on the assumption that any *a priori* biases will be similar across counties (Fig 7B).
