# Supplementary figures and images for: Downgrading disease transmission risk estimates using terminal importations
Source: PLoS Negl Trop Dis. 2019 Jun 14;13(6):e0007395. doi: 10.1371/journal.pntd.0007395 (PMC6594658; doi:10.1371/journal.pntd.0007395)

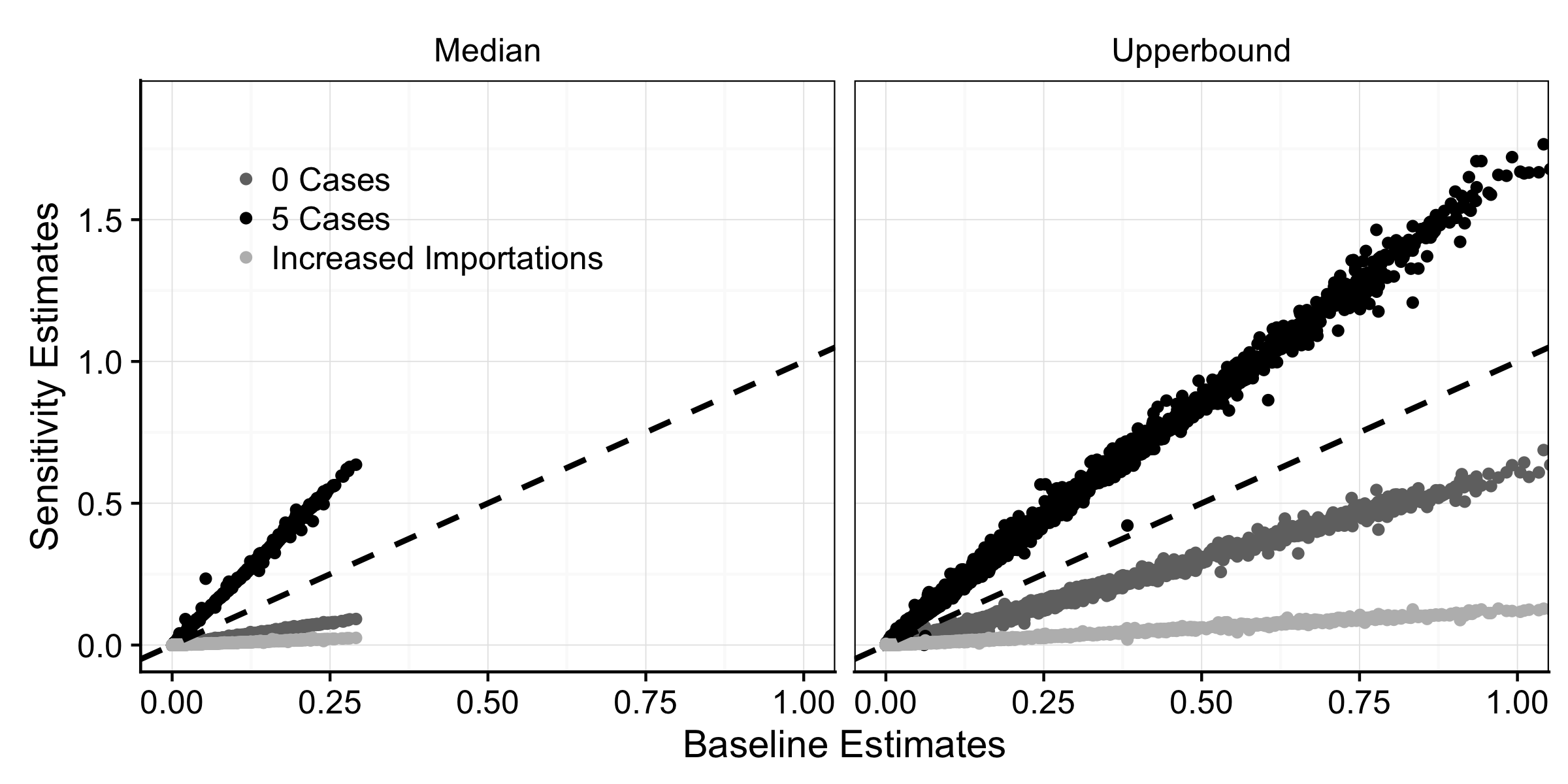

Supplement: S1 Fig — Each point indicates a county-month posterior R0 estimate under different estimation scenarios. The x-axis value for all points is determined by the baseline scenario where posterior R0 estimates consider only a single case detected in the November. The y-axis value is based on three sensitivity scenarios: (1) posterior estimates assuming no secondary transmission (dark grey), (2) five cases of secondary transmission in November (black), or (3) a single case of secondary transmission, but increased overall importations (light grey). Points falling above the black dashed line indicate that that a given scenario increases posterior R0 estimates compared to baseline estimates, and points below the line indicate the opposite. Estimates are compared for the median (left), and the 99th percentile (right) of the county-month distributions. Posterior R0 estimates increase if more secondary transmission is assumed, and decrease if less secondary transmission occurs, or if the absolute number of importations is increased. (TIFF) [file pntd.0007395.s002.tiff]

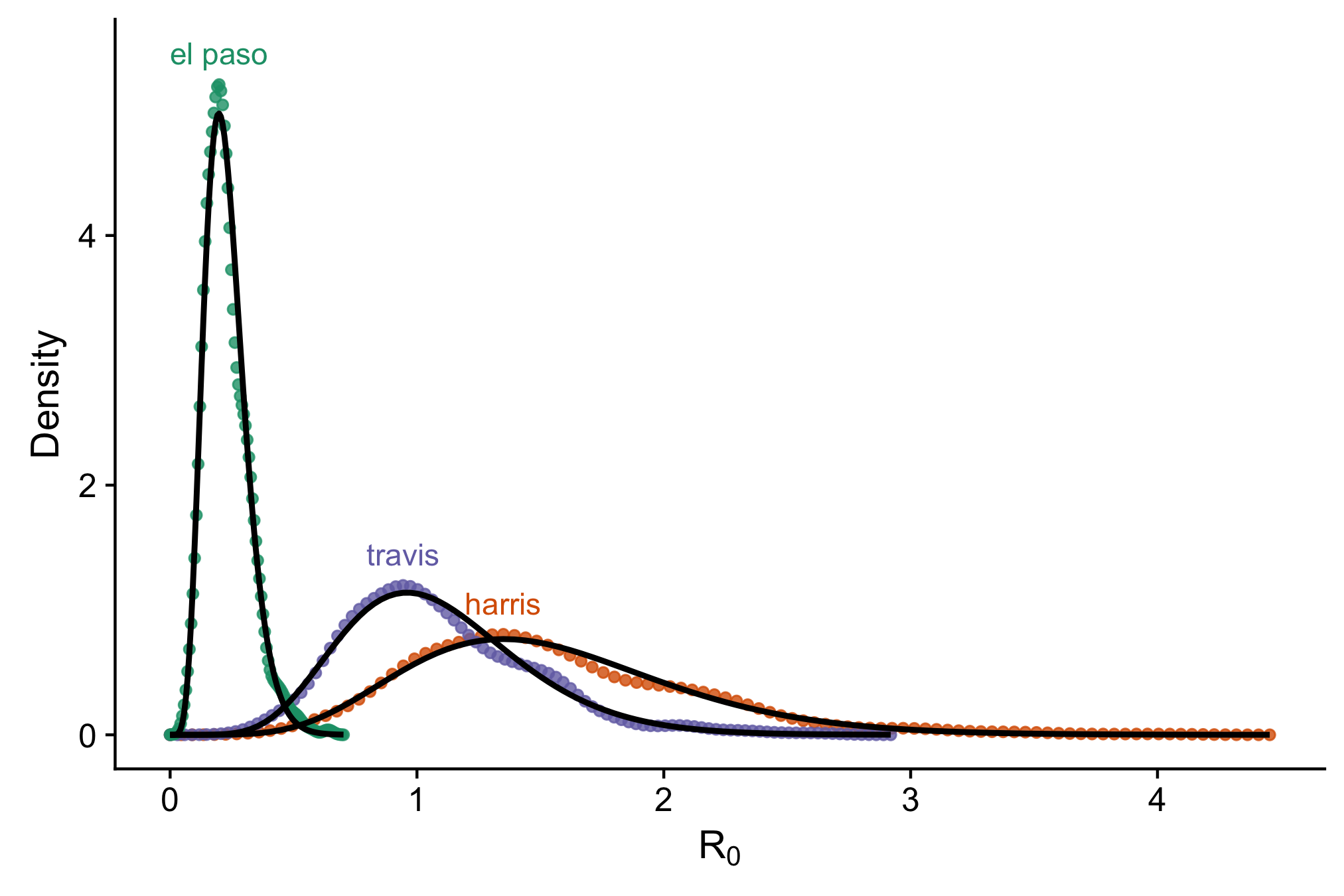

Supplement: S2 Fig — (TIFF) [file pntd.0007395.s003.tiff]

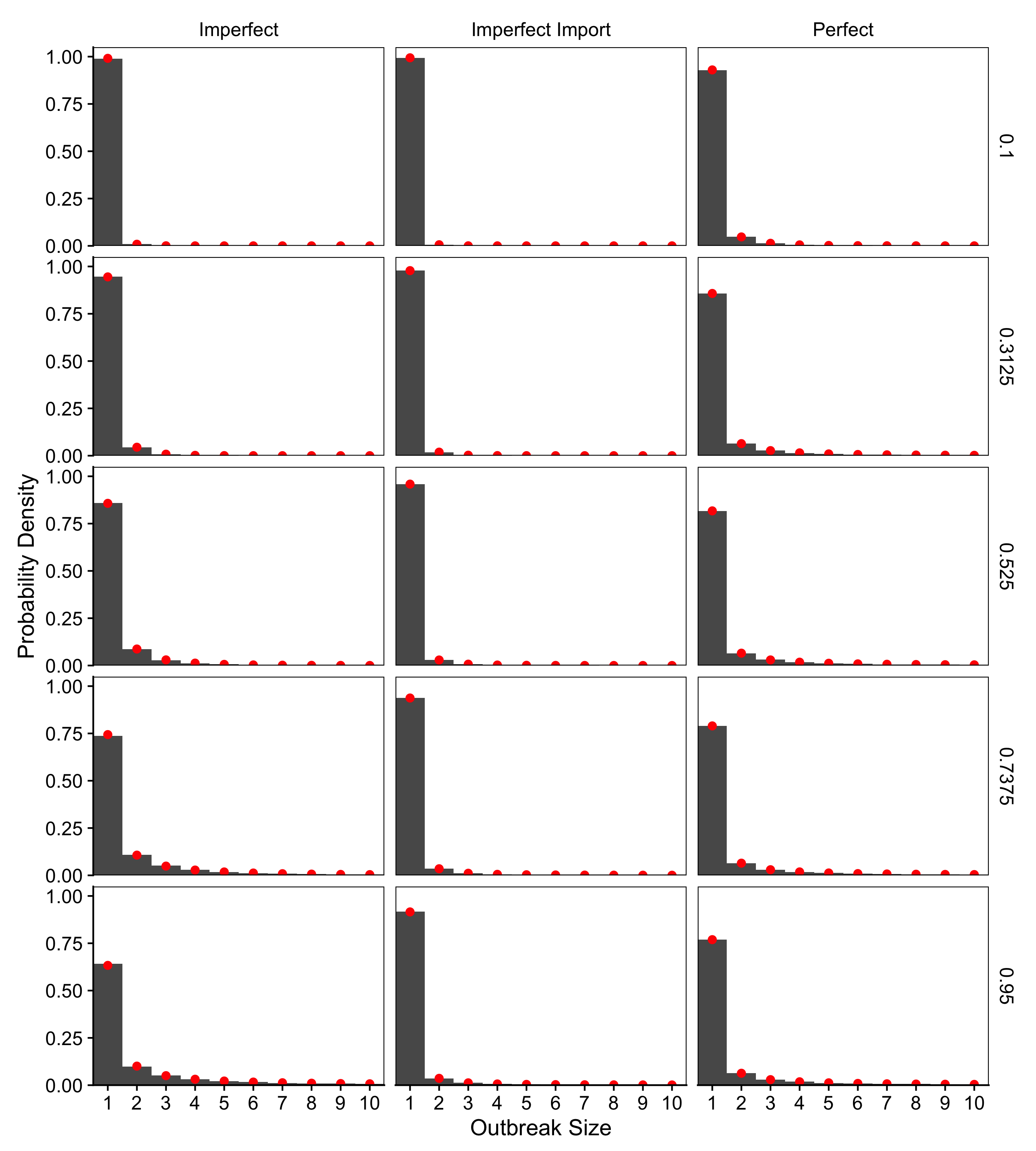

Supplement: S3 Fig — We compare the probability mass functions for the outbreak sizes for our simulations (bars) with the analytical expectation (red dots). Rows demonstrate four different transmission risk scenarios (R0), and columns describe three different scenarios: (1) where every case within a transmission chain is detected (Perfect), (2) where all cases are detected independently with a specific reporting rate (Imperfect), and (3) where all cases are detected independently with a specific reporting rate except for the index case which is always detected (Imperfect Import). The Imperfect Import probability mass function is the one used for all analyses in this article. All simulations are completed with a reporting probability of 0.0574, and k = 0.12. (TIFF) [file pntd.0007395.s004.tiff]

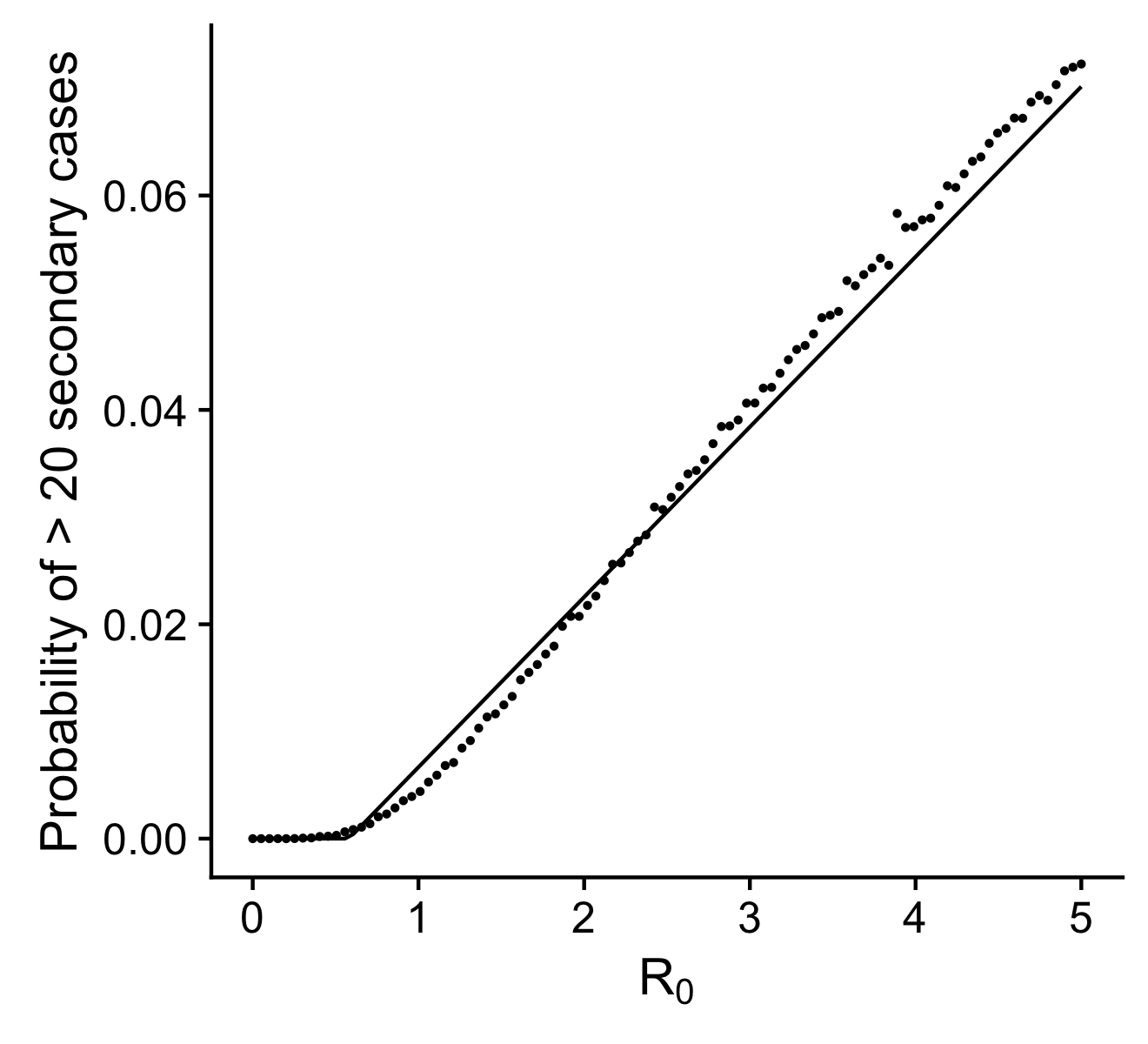

Supplement: S4 Fig — Probability of a single importation generating 20 secondary infections from (29) (Line), or using our assumed dispersion parameter and negative binomial distribution (Points). (TIFF) [file pntd.0007395.s005.tiff]

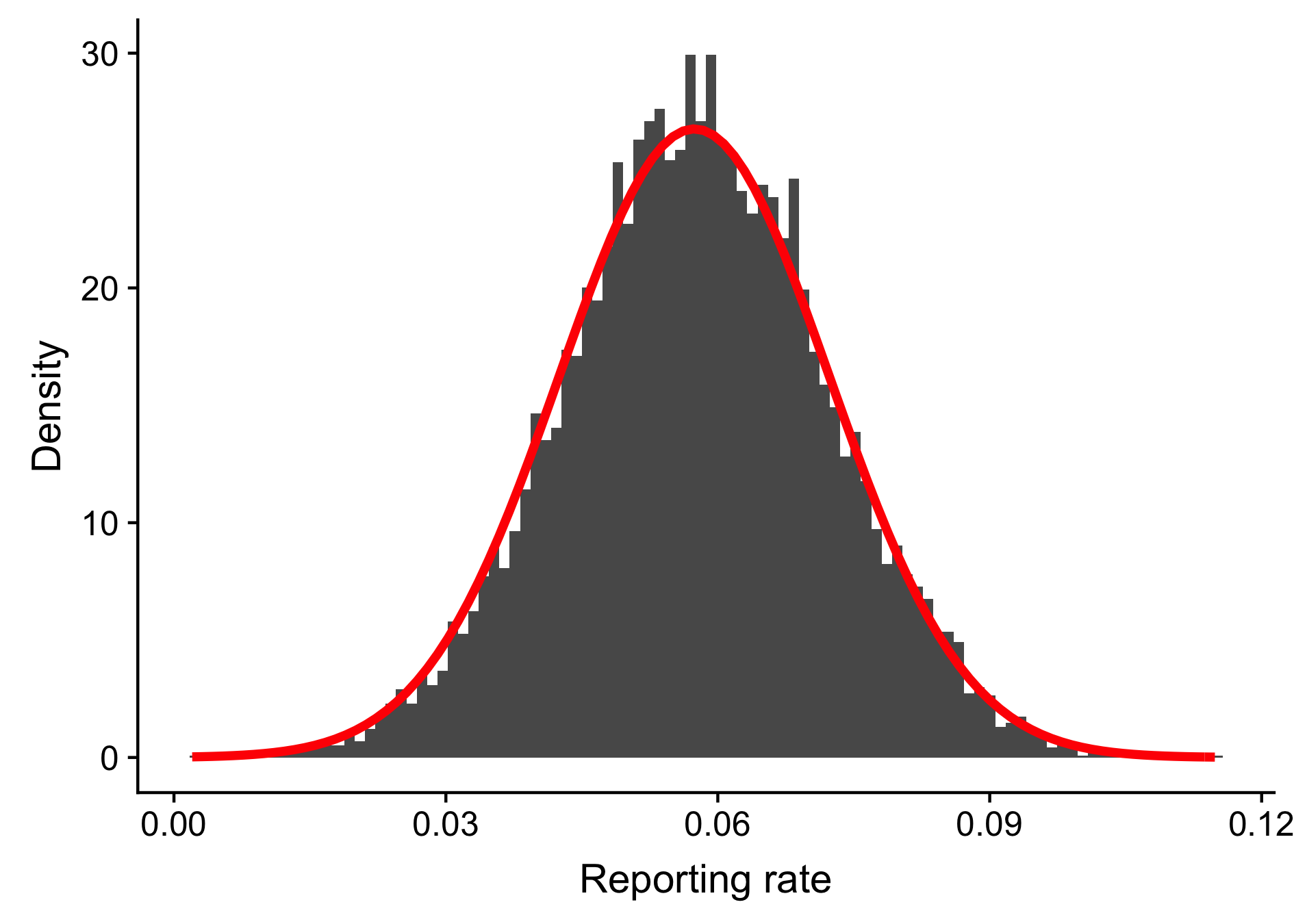

Supplement: S5 Fig — (TIFF) [file pntd.0007395.s006.tiff]

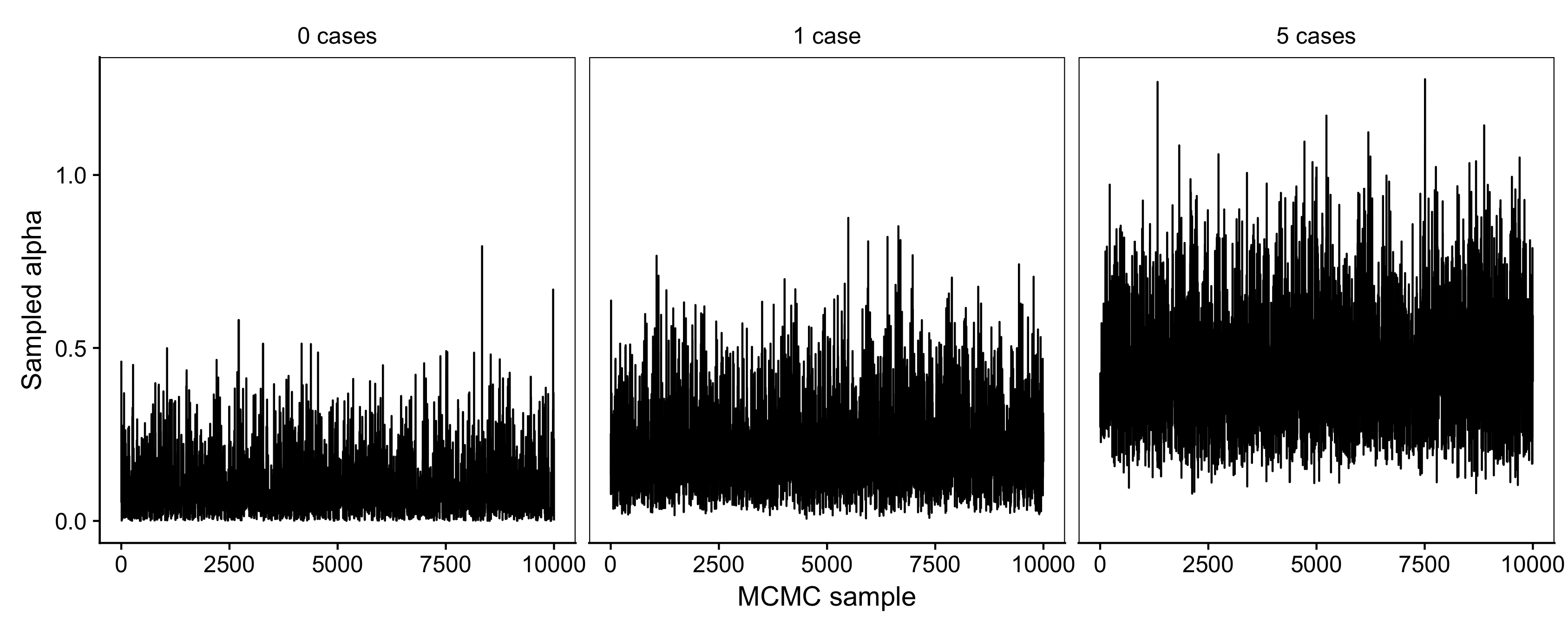

Supplement: S6 Fig — The plots detail the posterior distribution for alpha assuming the actual temperature from 2016 and 2017, and either 0, 1, or 5 detected cases in the year. (TIFF) [file pntd.0007395.s007.tiff]

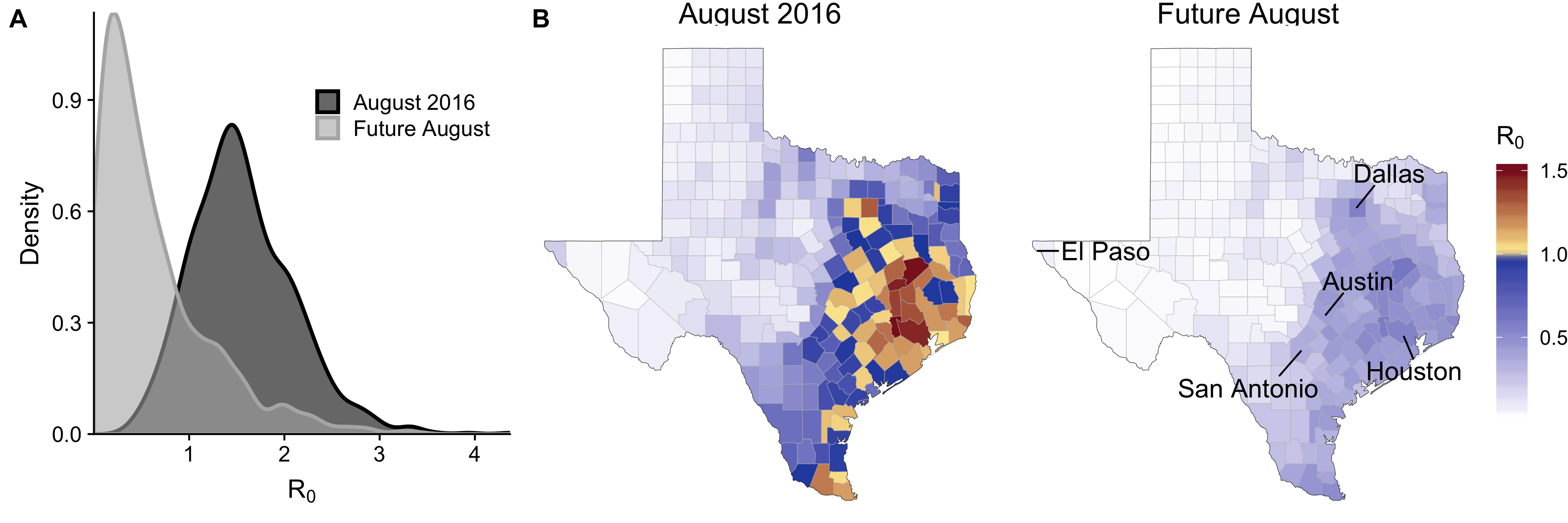

Supplement: S7 Fig — Consider a hypothetical scenario in which the first 15 terminal ZIKV importations into Texas arrive in Harris county (which includes Houston) during August 2016. (A) Estimated Harris county R0 for August 2016 a priori (dark grey) and after accounting for the 15 (light grey) terminal importations (Future August). These distributions are composed of 1,000 samples from the prior and posterior distributions (respectively). (B) Median R0 estimates for August before (August 2016) and following (Future August) the importation-based update. (TIFF) [file pntd.0007395.s008.tiff]

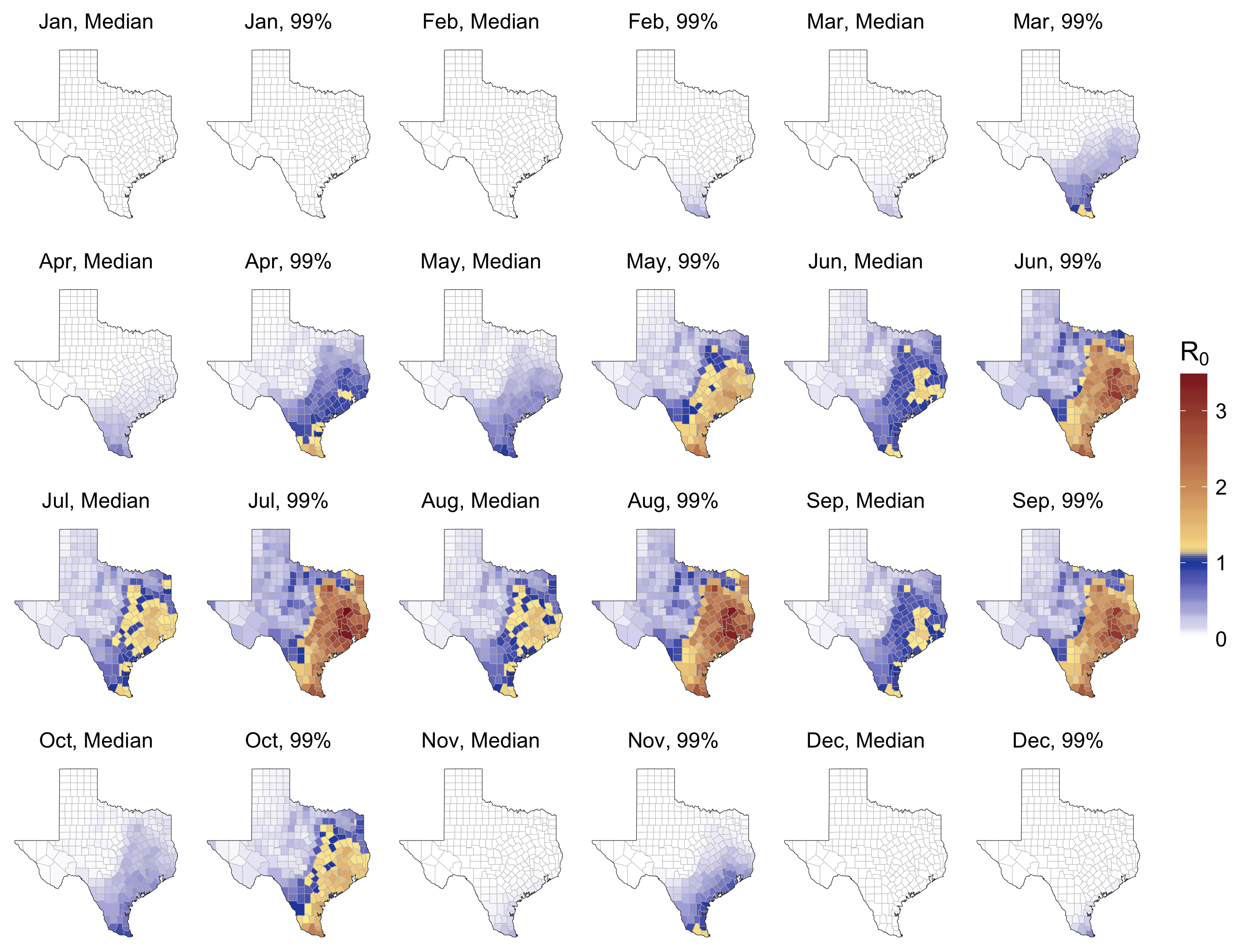

Supplement: S8 Fig — Median and 99 percentiles are shown for each County. Fill color indicates the estimated Median or 99 percentile estimate for that county for the given month, with counties showing yellow or red indicating their R0 is above one (labels). Estimates are made for each month based on the average monthly temperature for 2016. (TIFF) [file pntd.0007395.s009.tiff]

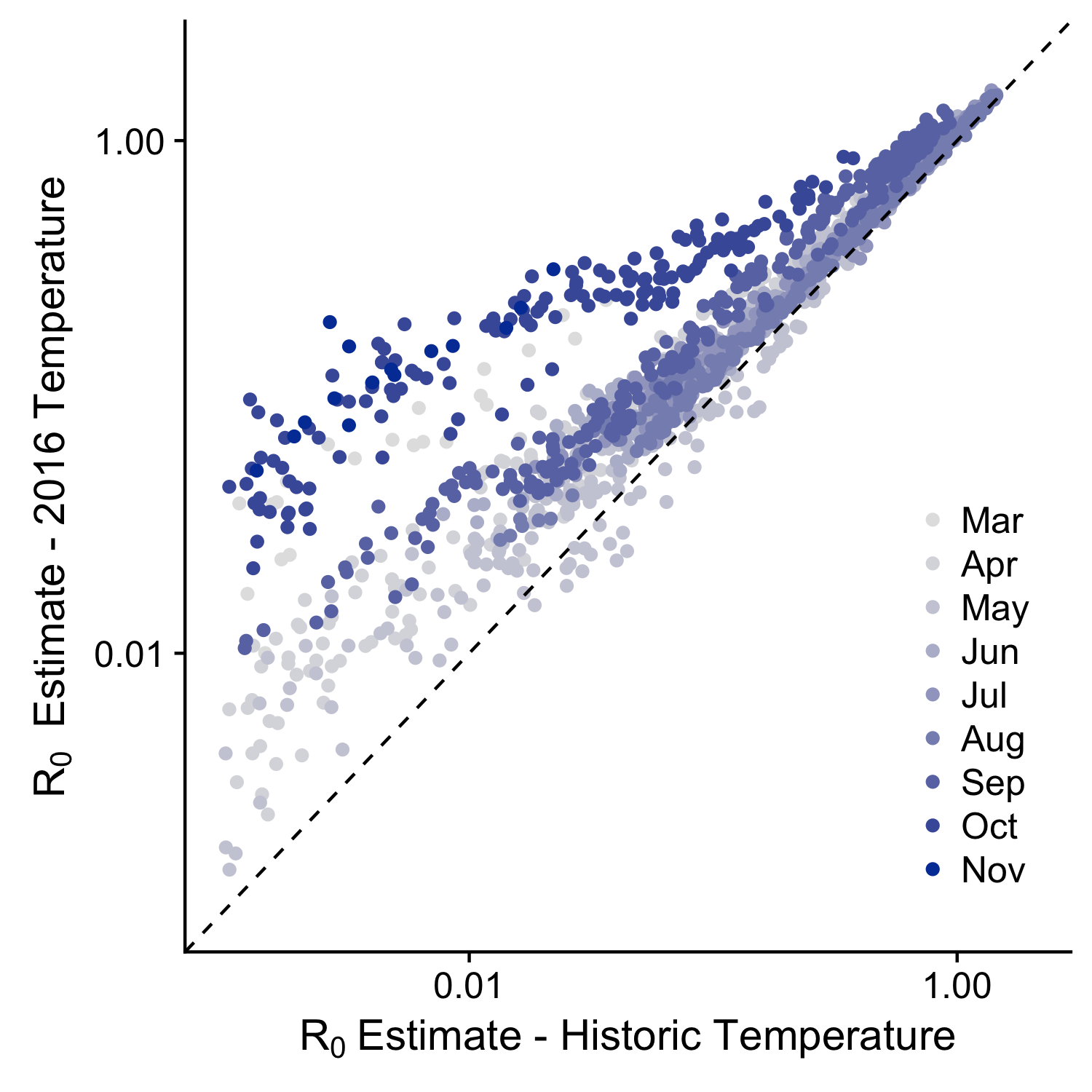

Supplement: S9 Fig — (TIFF) [file pntd.0007395.s010.tiff]

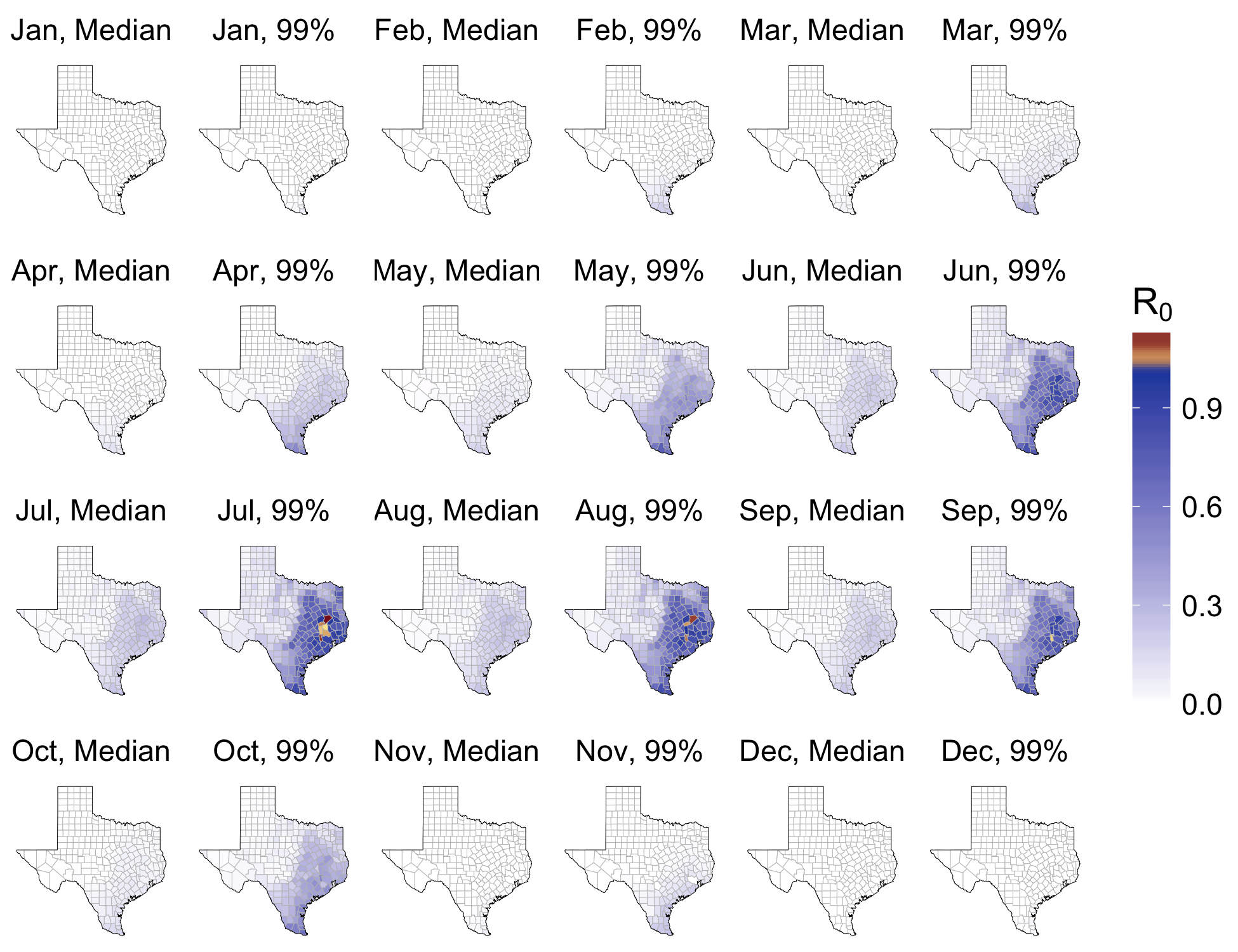

Supplement: S10 Fig — Fill color indicates the estimated Median or 99 percentile estimate for that county for the given month, with counties showing yellow or red indicating their R0 is above one (labels). Estimates are made using all importations through December of 2016, and assuming a single transmission event in both November and December. (TIFF) [file pntd.0007395.s011.tiff]

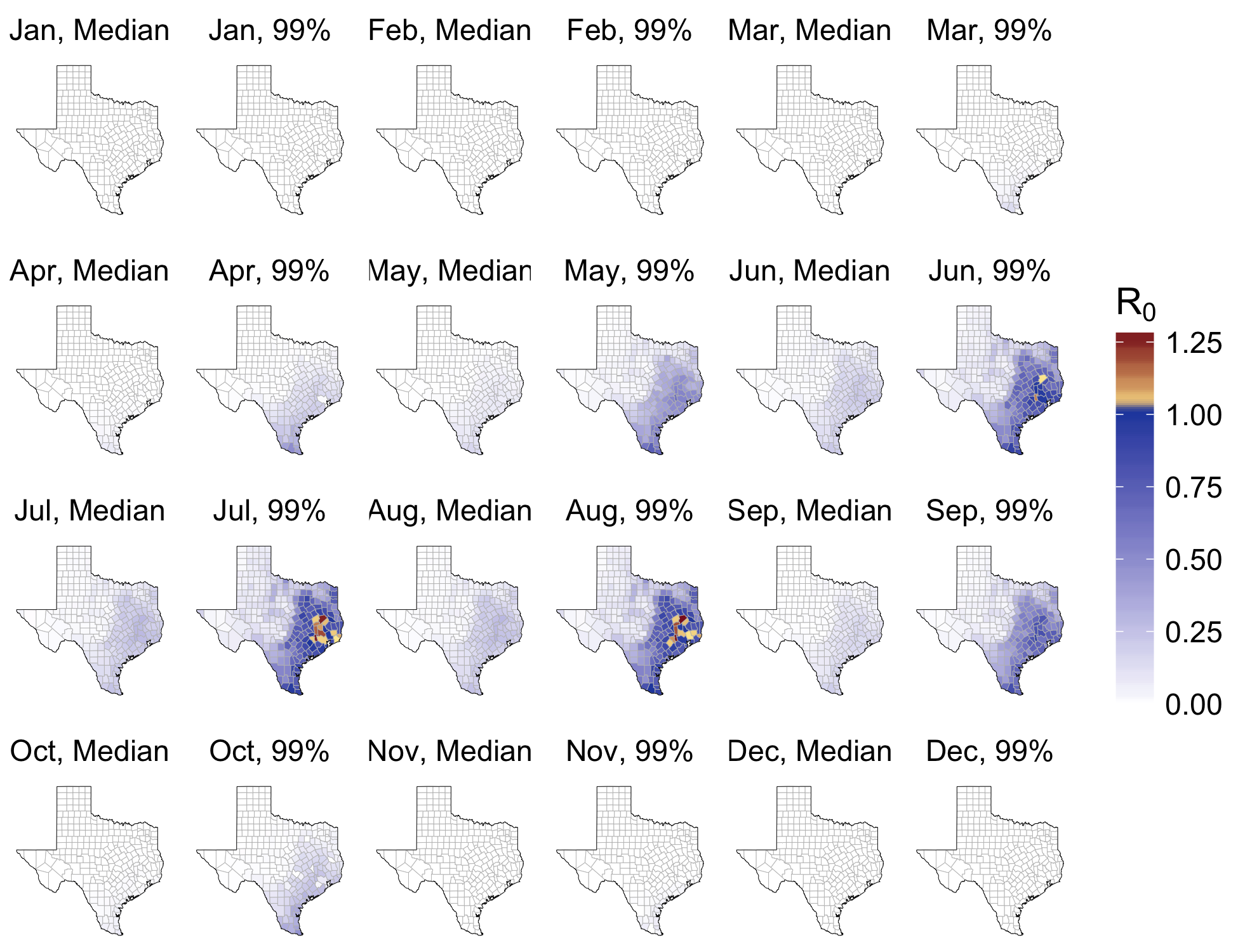

Supplement: S11 Fig — Fill color indicates the estimated Median or 99 percentile estimate for that county for the given month, with counties showing yellow or red indicating their R0 is above one (labels). Estimates are made using all importations through December of 2016, and assuming a single transmission event in November. (TIFF) [file pntd.0007395.s012.tiff]

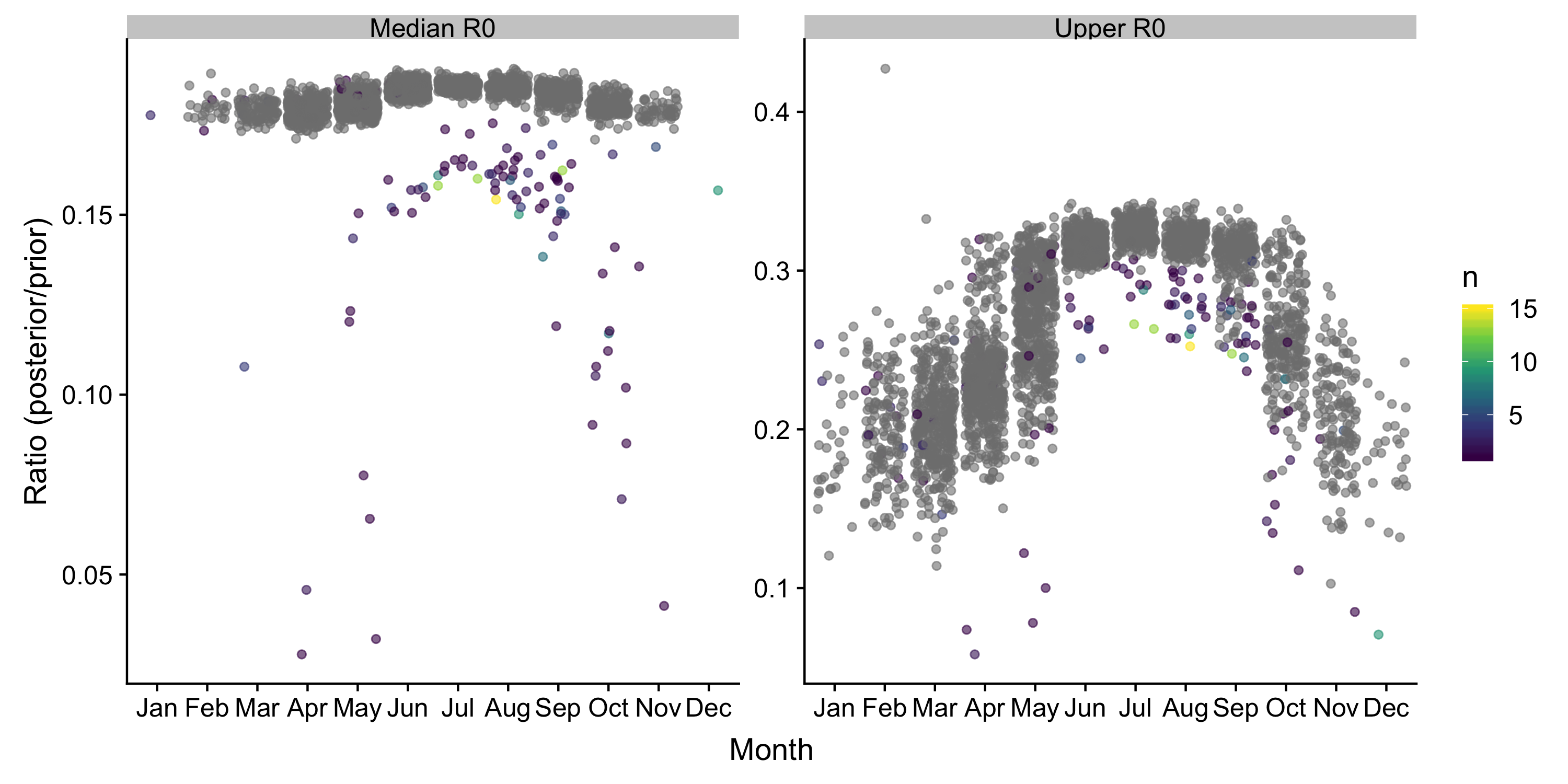

Supplement: S12 Fig — Each point corresponds to a specific county in the state, and the colors indicate the number of importations that the county experiences during the specific month. (TIFF) [file pntd.0007395.s013.tiff]

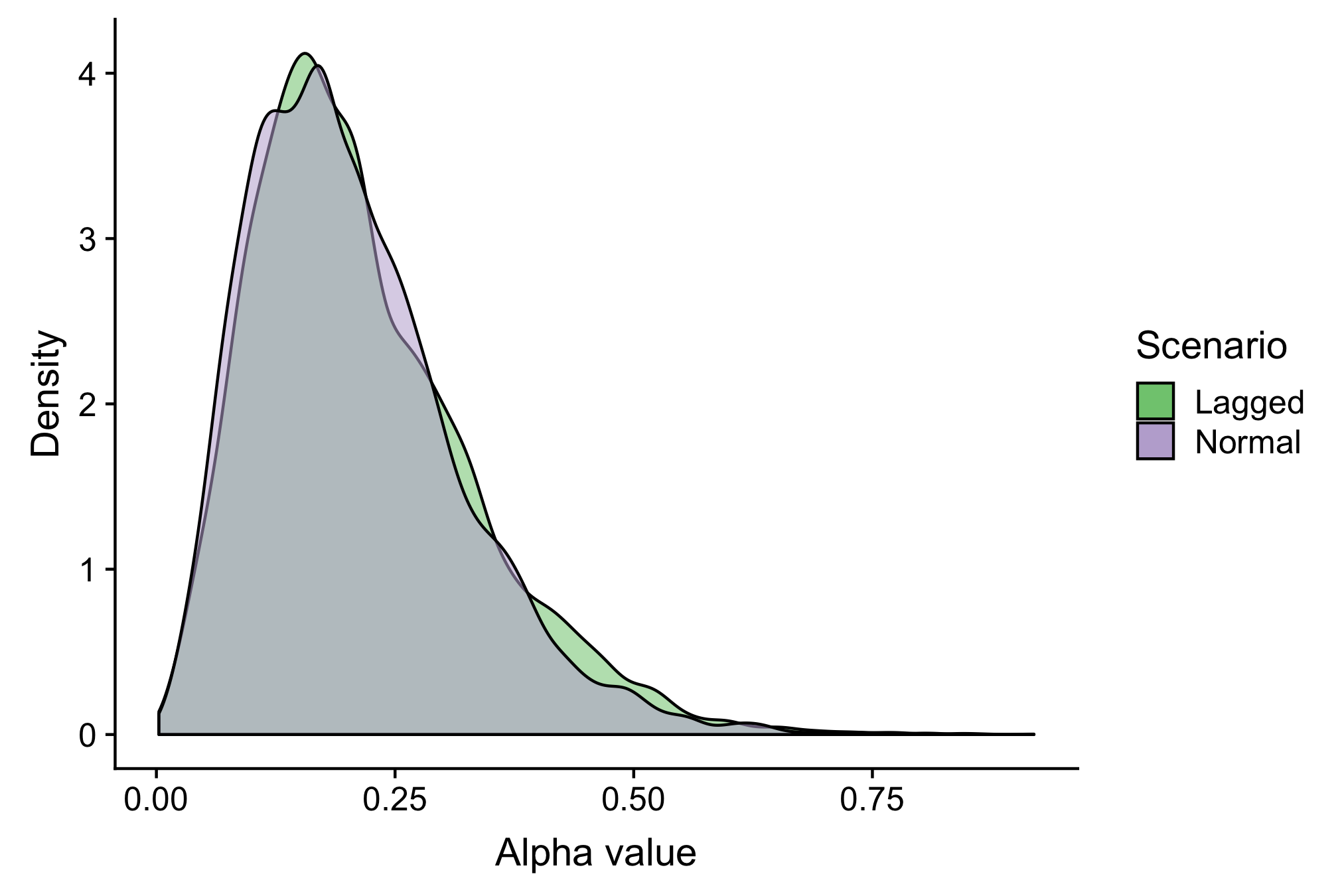

Supplement: S13 Fig — (TIFF) [file pntd.0007395.s014.tiff]

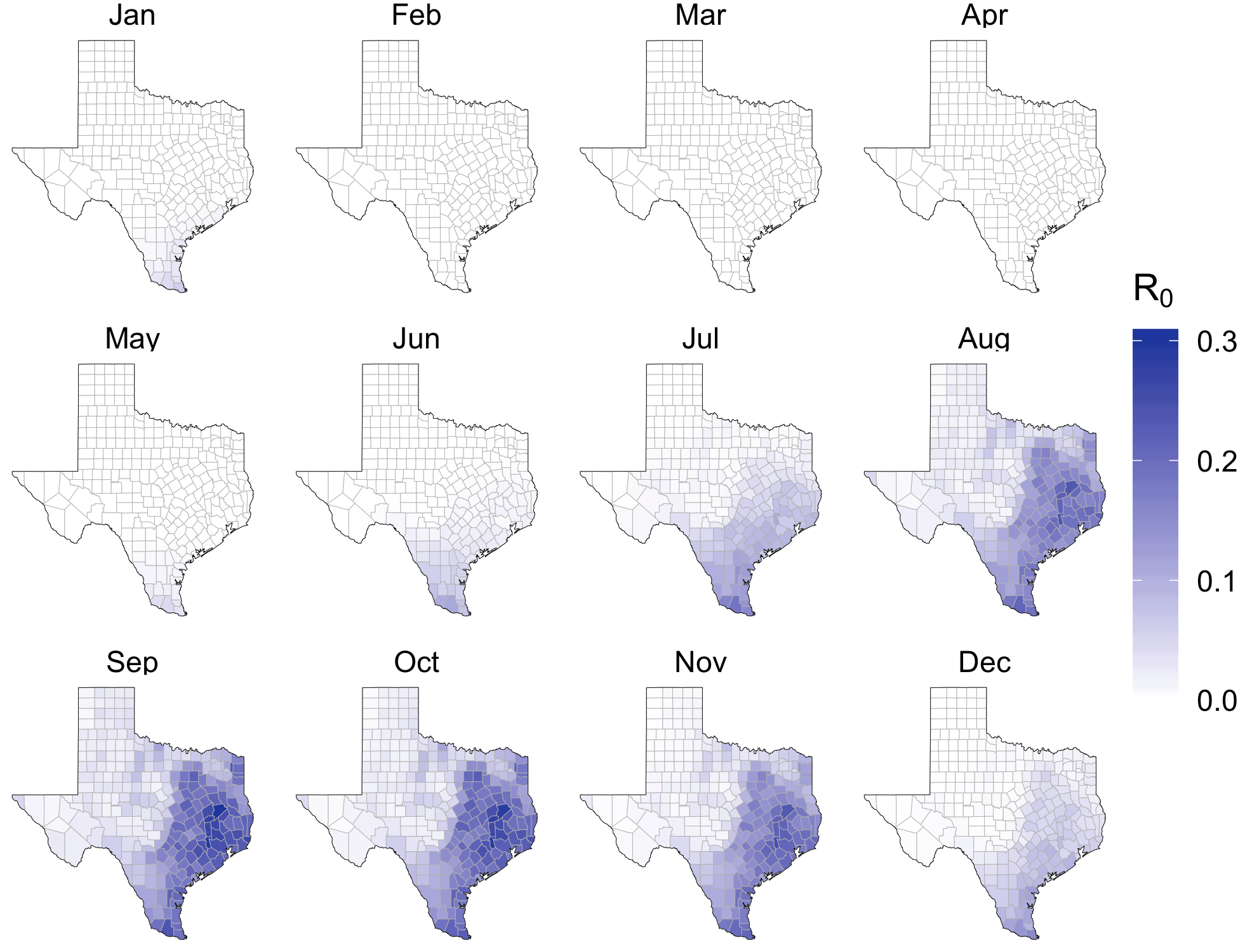

Supplement: S14 Fig — This assumes that all importations were terminal except for two autochthonous cases detected in Cameron County in late 2016, and shifts the prior transmission risk estimates by two months compared with the baseline scenario. (TIFF) [file pntd.0007395.s015.tiff]

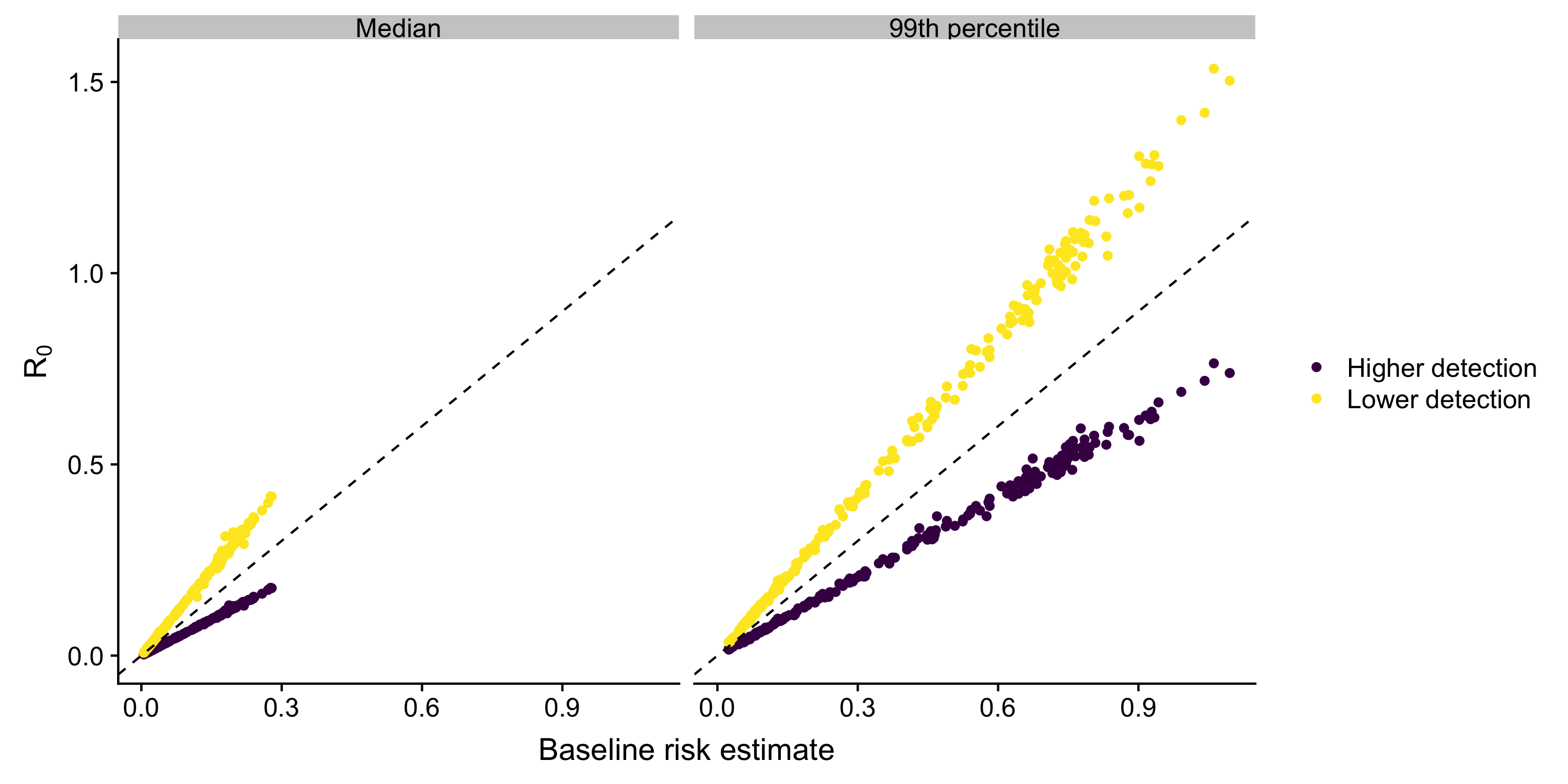

Supplement: S15 Fig — Dashed line indicates what the posterior estimates would be like if they matched the baseline posterior risk estimates. Colored points identify posterior estimates for different case detection probabilities. Yellow points are for a scenario where the probability for detecting cases was halved, and the purple points indicate estimates for when the probability was doubled. (TIFF) [file pntd.0007395.s016.tiff]
